# Hypothyroidism after hemithyroidectomy: a systematic review and meta-analysis

**DOI:** 10.1186/s13044-024-00200-z

**Published:** 2024-07-08

**Authors:** Dominic Cooper, Rajneesh Kaur, Femi E. Ayeni, Guy D. Eslick, Senarath Edirimanne

**Affiliations:** 1 The University of Sydney School of Medicine, Sydney, Australia; 2 Nepean Institute of Academic Surgery, The University of Sydney School of Medicine, 62 Derby St, Kingswood, Sydney, NSW 2750 Australia; 3 The University of Sydney School of Medicine, Nepean Clinical School, Sydney, Australia

**Keywords:** Hypothyroid, Hypothyroidism, Hemithyroidectomy, Thyroid lobectomy, Thyroxine

## Abstract

**Background:**

The incidence of hypothyroidism following hemithyroidectomy and risk factors associated with its occurrence are not completely understood. This systematic review investigated the incidence and risk factors for hypothyroidism, thyroxine supplementation following hemithyroidectomy as well as the course of post-operative hypothyroidism, including the time to hypothyroidism and incidence of transient hypothyroidism.

**Methods:**

Searches were conducted in MEDLINE, EMBASE, Scopus, and Cochrane library for studies reporting the incidence of hypothyroidism or thyroxine supplementation following hemithyroidectomy.

**Results:**

Sixty-six studies were eligible for inclusion: 36 reported risk factors, and 27 reported post-operative course of hypothyroidism. Median follow-up was 25.2 months. The pooled incidence of hypothyroidism was 29% (95% CI, 25-34%; *P*<0.001). Transient hypothyroidism occurred in 34% of patients (95% CI, 21-47%; *P*<0.001). The pooled incidence of thyroxine supplementation was 23% (95% CI, 19-27%; *P*<0.001), overt hypothyroidism 4% (95% CI, 2-6%, *P*<0.001). Risk factors for development of hypothyroidism included pre-operative thyroid stimulating hormone (TSH) (WMD, 0.87; 95% CI, 0.75-0.98; *P*<0.001), TSH ≥ 2 mIU/L (RR, 2.87; 95% CI, 2.43-3.40; *P*<0.001), female sex (RR, 1.19; 95% CI, 1.08-1.32; *P*=0.007), age (WMD, 2.29; 95% CI, 1.20-3.38; *P*<0.001), right sided hemithyroidectomy (RR, 1.35; 95% CI, 1.10-1.65, *P*=0.003), the presence of autoantibodies anti-TPO (RR, 1.92; 95% CI, 1.49-2.48; *P*<0.001), anti-Tg (RR, 1.53; 95% CI, 1.40-1.88; *P*<0.001), and Hashimoto’s thyroiditis (RR, 2.05; 95% CI, 1.57-2.68; *P*=0.001).

**Conclusion:**

A significant number of patients will develop hypothyroidism or require thyroxine following hemithyroidectomy. An awareness of patient risk factors and postoperative thyroid function course will assist in counselling patients on their risk profile and guiding management.

**Supplementary Information:**

The online version contains supplementary material available at 10.1186/s13044-024-00200-z.

## Introduction

Hemithyroidectomy is commonly used for the management of benign unilateral thyroid conditions and low risk differentiated malignancy. In most cases the remaining thyroid gland can compensate and maintain normal thyroid function in the absence of one thyroid lobe, but a minority of patients will develop hypothyroidism following surgery [1]. Hypothyroidism is readily managed with thyroxine treatment. Thyroxine therapy though have been shown to reduce the risk of thyroid nodules by inhibiting nodule growth [2], but it is also associated with adverse cardiovascular, and musculoskeletal effects [3, 4], and demands regular monitoring and follow up, meaning it represents an important complication for many patients. The incidence of hypothyroidism following hemithyroidectomy shows heterogeneity in the literature, with reported incidence ranging from 9.5% to 64.2% [5, 6]. This may be explained by many factors, such as regional differences in iodine status and baseline thyroid function, definition of hypothyroidism, sample size, follow up protocol and duration, and patient characteristics such as age, pre-operative thyroid stimulating hormone (TSH) and sex [7–9]. Some of these factors can be assessed in the pre-operative period to advise patients on their individualised risk of hypothyroidism, which can improve decision making and thus help in the identification of high-risk patients for closer follow up and assessment.

A meta-analysis was published in November 2020 on hypothyroidism after hemithyroidectomy [10]. That study found the prevalence of hypothyroidism to be 29.9%, as well as several risk factors for its development.

Our report aimed to build on the previous analysis in several regards. Firstly, that study did not explore the risk of overt hypothyroidism. Overt hypothyroidism reflects a progression from biochemical hypothyroidism and has significant health consequences that necessitates treatment with thyroxine [11]. Further, our study explored several previously unreported risk factors, including the risk of older age and autoantibodies anti-TPO and anti-Tg.

Finally, the postoperative course of thyroid function remains unclear. Thyroid function following hemithyroidectomy has been observed to decline in the short term (≤ 6 months) before subsequently recovering [12], with many patients attracting only a transient diagnosis of hypothyroidism [13–15]. In the absence of suitable follow up, this may lead to overdiagnosis of hypothyroidism in the short term after surgery and overtreatment of potentially self-resolving cases of hypothyroidism.

The aim of this study was to develop an updated systematic review and meta-analysis of the literature to determine the incidence of hypothyroidism after hemithyroidectomy, analyse risk factors associated with the development of hypothyroidism, and clarify the natural course of post-surgical hypothyroidism, including the time to hypothyroidism, post-operative TSH recovery, as well as the incidence of transient hypothyroidism to improve clinical decision making.

## Methods

### Literature search strategy

Literature searches were conducted through MEDLINE, EMBASE, Scopus, and Cochrane library databases. The search terms included “hemithyroidectomy” OR “hemi thyroidectomy” OR “hemi-thyroidectomy” OR “lobectomy” OR “thyroid lobectomy” AND “hypothyroid*” OR “levothyroxine” OR “thyroxine” OR “thyroid hormone”. The search was limited to the English language. There was no limit on publication date. The search was current until the 18^th^ of May 2023. Reference lists of included papers were reviewed for any further relevant papers.

### Eligibility criteria

Studies reporting hypothyroidism or thyroxine supplementation following hemithyroidectomy or thyroid lobectomy were eligible for inclusion. Where studies reported on thyroxine supplementation and not hypothyroidism it was assumed that the indication for thyroxine initiation was hypothyroidism unless otherwise specified. Studies that empirically treated with thyroxine supplementation were excluded. Where studies included multiple surgical types, such as subtotal, partial, or total thyroidectomies, only the hemithyroidectomy group was included. No restriction was placed on the indication for surgery, with both benign and malignant indications included. Papers that did not explicitly include preoperative thyroid status were included. Where multiple studies used overlapping data, the first publication was used. Only full text articles were considered for inclusion. Meeting abstracts and unpublished data were excluded. Only papers in the English language were included.

### Study selection and data extraction

All papers were screened by two reviewers (DC and SE) by title, then abstract and full text. The included papers were then analysed, with data extracted by a single reviewer (DC). The data was extracted into a predefined spreadsheet. Data extracted included (i) study characteristics (author, year of publication, country of origin), (ii) cohort characteristics (age, sex, cohort number), (iii) surgical information (indication for surgery, side of hemithyroidectomy, benign or malignant pathology, thyroid comorbidities), (iv) biochemistry (pre-operative TSH, thyroid autoantibodies), and (v) postoperative follow up (hypothyroid incidence, transient hypothyroid incidence, time of hypothyroid diagnosis, follow up duration, protocol for thyroid status measurements, and postoperative thyroid hormone supplementation incidence and indication).

### Quality assessment

The studies were assessed using a modified Newcastle Ottawa Scale for cohort studies (Sup. Table 1). The assessment was carried out independently by one primary reviewer (DC) and reviewed by a second independent reviewer (SE). Differences were resolved by unanimous agreement.

### Statistical analysis

A meta-analysis of the pooled data was performed using Review Manager 5.4 [16]. Dichotomous variables were reported using a risk ratio (RR), and continuous variables by a weighted mean difference (WMD). Statistical significance was defined as *P* < 0.05. The Mantel-Haenszel random effects model was used following consideration of the study heterogeneity. Heterogeneity was assessed using the Chi^2^ test and reported with the I^2^ statistic, with I^2^ <50%, 50-75%, >75% used to indicate low, moderate, and high degrees of heterogeneity respectively. Forest plots were constructed for each outcome of interest. Funnel plot symmetry was used to assess publication bias.

## Results

### Literature search

**Fig. 1:**
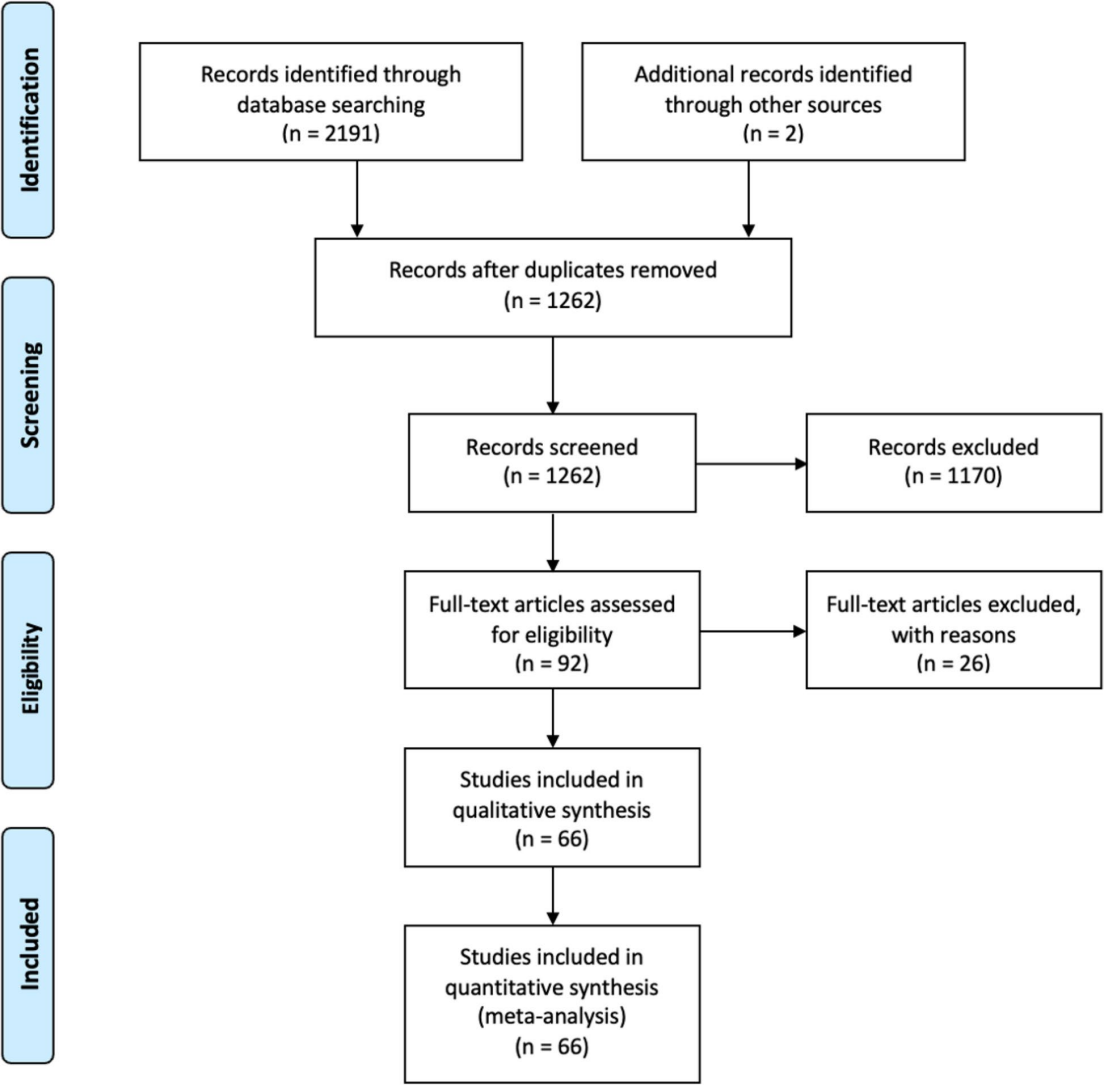
PRISMA diagram illustrating selection of studies

The search protocol produced 1262 non duplicate articles. Following screening by title and abstract, 92 papers remained. A further 26 papers were excluded on full text review for the following reasons: non-English language (*n*=3), reported incidence of hypothyroidism or thyroxine supplementation (*n*=3), no full text available (*n*=5), empirical thyroxine treatment (*n*=8), conference article or poster presentation (*n*=5), duplicate patient cohorts (*n*=2). 66 articles were included in the review. The PRISMA flowchart is represented in Fig. 1

### Quality Assessment results

The 66 cohort studies were assessed using a modified Newcastle Ottawa scale for cohort studies (Sup.Table 1). Of these, 42 studies scored ≥ 5/6 and were considered high quality.

### Study characteristics

The study characteristics are outlined in Table 1*.* Sixty-six studies were included with a total of 13,546 patients [5, 6, 9, 13, 15, 17–77]. Study sizes ranged from 14 to 1240 participants. Publication years ranged from 1986 to 2022. The postoperative incidence of hypothyroidism ranged from 0 to 66.2%. The mean age ranged from 13.65 to 53 years, with a median of 47.7 years. Females accounted for the majority of patients in sixty-five studies.

**Table 1:** Study characteristics

**Article**	Patient Number	Hypothyroid, No. (%)	Age, *years,* mean (SD)	Sex, female, No. (%)	Follow up duration, *months,* (range)	Definition of hypothyroidism	Interval to hypothyroidism from intervention, *months*	Thyroid function follow-up protocol
**Abraham, C. R., et al. (2022)** [17]	170	63 (37.1)	47.0 (14.1)	140 (82.4)	22.7	TSH > 4.5 mIU/L	NR	NR
**Ahn, D., et al. (2016)** [7]	405	226 (55.8)	47.4	345 (85.2)	56.4 (12-105)	TSH > 4.5 mIU/L	4.3	TF measured 1–3 months after surgery. If normal, follow-up thyroid function measurements were performed every 6–12 months. If subclinical hypothyroidism was identified, thyroid function measurement was performed at 3 months and repeated every 3–6 months
**Akkari, M., et al. (2014)** [18]	14	3 (21.4)	NR	NR	45	NR	5.6	NR
**Alsaleh, N., et al. (2021)** [19]	38	13 (34.2)	42 (14)	25 (65.8)	24	TSH > 4.8 μIU/mL	NR	TFT measured at least 6 weeks postoperatively
**Al-Shalhoub, A. K. and S. Al-Dhahri (2017)** [20]	83	37 (44.6)	38	67 (80.7)	12	TSH > 5 mIU/L	NR	The first postoperative thyroid function follow-up test was performed at different time points; whereas 40 patients (48.2%) had their first postoperative thyroid function test at 3 months, 26 patients (31.13%) had it at 6 months, 6 patients (7.2%) at 9 months, nine patients (10.8%) at 12 months and two patients (2.4%) at 18 months
**Antunes, C. M. and A. Taveira-Gomes (2013)** [21]	109	40 (36.7)	48.68 (14.93)	NR	60	NR	NR	NR
**Attaallah, W., et al. (2015)** [22]	259	56 (21.6)	43.1 (12.2)	195 (75.3)	31 (6-126)	NR	NR	NR
**Balentine, C. J., et al. (2013)** [23]	17	8 (47.1)	52.8 (16.5)	15 (88.2)	NR	NR	NR	NR
**Baran, J. A., et al. (2021)** [24]	110	31 (28.2)	14.9 (2.5)	94 (85.5)	12 (1-62)	TSH > 4.5 mIU/L	1.7	NR
**Barczynski, M., et al. (2010)** [25]	72	17 (23.6)	40.1 (10.2)	62 (86.1)	60	NR	NR	All the patients were evaluated every 3 months during the first year and every 12 months for the following years up to completion of the planned 60-month follow-up. The biochemical evaluation consisted of serum values of fT3, fT4, and TSH
**Bauer, P. S., et al. (2013)** [26]	420	79 (18.8)	50.7	327 (77.9)	46.1	NR	NR	NR
**Beisa, V., et al. (2015)** [27]	109	20 (18.4)	NR	91 (83.5)	12	TSH > 4 mIU/L	NR	Postoperative TSH and LT3 and LT4 tests were performed 2, 6, and 12 months after the surgery
**Beisa, V., et al. (2011)** [28]	216	48 (22.2)	NR	194 (89.8)	20	TSH > 4 mIU/L	NR	A postoperative TSH test was performed 2, 6 and 20 months after the surgery
**Berglund, J., et al. (1998)** [29]	26	0 (0)	48.5	20 (76.9)	12	NR	NR	Serum concentrations of thyroxine, triiodothyronine (T3) and thyroid stimulating hormone (TSH) were measured preoperatively and 1, 3, 6, 12 months postoperatively
**Buehler, L. A., et al. (2021)** [13]	210	96 (45.7)	51.1 (15.3)	NR	50 (12-59.9)	TSH > 4.2 mIU/L	NR	NR
**Cao, Z., et al. (2022)** [30]	378	110 (29.1)	NR	280 (74.1)	NR	TSH > 4.34 μIU/ml	3.47	During the followup period, patients underwent thyroid function assessments at 1, 3, 6, and 12 months postoperatively, then every six months after that. However, when patients complained the clinical symptoms related to thyroid dysfunction or had suspicious TFT results, TFTs were performed at any time
**Chen, J., et al. (2019)** [31]	43	11 (25.6)	13.65 (3.04)	34 (79.1)	28	TSH > 3.9 μIU/mL	4.1	TSH measured at least 6 weeks postoperatively
**Cheung, P., et al. (1986)** [32]	103	13 (12.6)	39	80 (77.7)	36	NR	NR	TSH and fT4 measured at 6 months, 1, 2, and 3 years postoperatively
**Chidambaranathan, N., et al. (2021)** [33]**)**	128	26 (20.3)	36.5	103 (80.5)	27.5 (0-96)	NR	6.5	The first TFT was done on most occasions at around 3–6 months, and subsequently thereafter, it was repeated every 6–12 months
**Cho, J. S., et al. (2011)** [34]	123	26 (21.2)	NR	87 (70.7)	35.7 (06-80)	TSH > 6 mIU/L	NR	Patients were followed up at 3 to 6 months intervals during the first two postoperative years, and annually thereafter
**Cho, M. J., et al. (2021)** [35]	29	6 (20.7)	40 (2)	23 (79.3)	12	TSH > 4.1 μIU/ml	NR	TSH was measured before surgery, and 3 months and 1 year after surgery
**Chong, S. S., et al. (2019)** [36]	101	23 (22.8)	48	83 (82.2)	29.3	TSH > 4.2 μIU/mL	NR	TSH, total T4, T3 were measured 2 months after surgery
**Chotigavanich, C., et al. (2016)** [37]	100	27 (27)	43.6 (12.2)	93 (93)	NR	TSH > 4 μIU/mL	NR	TFT was performed six weeks after surgery
**Chu, K. K. W. and B. H. H. Lang (2012)** [38]	263	38 (14.5)	NR	204 (77.6)	21 (3– 62)	TSH > 5.5 mIU/L	3	Postoperative serum TSH was monitored regularly at 2 weeks, 3 months, 6 months, and yearly after surgery until the patient was lost to follow-up evaluation
**De Carlucci Jr, D., et al. (2008)** [39]	186	61 (32.8)	45 (14)	163 (87.6)	29 (6-108)	TSH > 5.5 mIU/L	NR	TSH and fT4 measured postoperatively, 4 to 8 weeks after surgery
**Dou, Y., et al. (2020)** [8]	190	77 (40.5)	40.4 (10.4)	140 (73.7)	36 (20-36)	TSH > 5.92 mIU/L	NR	TFT in the first month, every three months during the first postoperative year, and then every six months during the second and third years
**Ergul, Z., et al. (2014)** [40]	50	8 (16)	42.3 (12.7)	36 (72)	25.2 (10-43)	TSH > 4.5 μIU/mL	NR	Follow-up with thyroid function tests on the first month and then once every three months, as well as ultrasonography controls once a year were performed postoperatively
**Ha, T. K., et al. (2019)** [41]	175	87 (49.7)	45.2 (12.2)	148 (84.6)	7.5 (1-12)	NR	NR	TFTs were performed routinely at 1, 3, 6, and 12 months after lobectomy
**Ito, M., et al. (2015)** [42]	150	56 (37.3)	NR	116 (77.3)	18	TSH > 5 mIU/L	NR	Thyroid function tests were performed 1 month after surgery and every 3–6 months thereafter
**Johner, A., et al. (2011)** [12]	117	25 (21.4)	49.6	98 (83.8)	NR	TSH > 5.5 mIU/L	NR	TSH at 6 weeks or 3 months, and 6 and 12 months postoperatively. Followed 6 monthly or annually depending on category of TSH level
**Kim, C. J., et al. (2020)** [14]	252	98 (38.9)	NR	47 (18.7)	38.3 (3-104)	TSH > 4.05 mIU/L	NR	TSH and fT4 levels were checked postoperatively at 1 week, 1-3 months, 6 months, and annually and/or biannually thereafter
**Kim, S. Y., et al. (2020)** [43]	256	169 (66)	43.79 (10.92)	204 (79.7)	64.5	TSH > 4.7 mIU/L	NR	TFT at the time of outpatient visit every 6 months for the 1st year, with an annual follow-up thereafter
**Koh, Y. W., et al. (2008)** [44]	136	58 (42.7)	42.7 (11.7)	115 (84.6)	22.2	TSH > 4 mIU/L	NR	TSH and T4 at 1, 6 and 12 months
**Kristensen, T. T., et al. (2014)** [45]	28	8 (28.6)	49	22 (78.6)	12	NR	3.9	TSH and thyroid hormones at 1, 3, 6 and 12 months
**Lang, B. H., et al. (2017)** [46]	150	44 (29.3)	NR	125 (83.3)	53.5	TSH > 4.78 mIU/L	3.2	Postoperative TSH and FT4 were checked at 1, 3, 6, and 12 months, then every 6 months thereafter
**Lankarani, M., et al. (2008)** [47]	45	8 (17.8)	NR	NR	12	NR	NR	During the first three months after surgery, all patients were visited monthly, then 2 monthly and then on a 3 monthly basis. In each visit TFT of each patient was repeated and registered
**Latoo, M. A., et al. (2020)** [48]	50	18 (36)	35.58 (10.226)	38 (76)	12	TSH > 4.5 mIU/L	3.9	The first TFT measured 1 month after surgery. If normal, follow-up measurements of thyroid function were performed at 6 months and 12 months. If subclinical hypothyroidism was identified at any time during the follow-up period, thyroid function measurement was performed 3 months after that point and was repeated every 3 months
**Lee, D. Y., et al. (2015)** [49]	276	65 (23.6)	49	196 (71)	28.8 (3.67-105)	NR	3.2	TSH levels at 1 week, 1 month, 4 months, 1 year, and 2 years
**Lee, S. J., et al. (2021)** [50]	353	193 (54.7)	46.4 (12.3)	258 (73.1)	31.4	TSH > 4.2 μIU/ml	5.5	TSH and fT4 levels were measured at 1 week, 1 month, and 3, 6, and 12 months, and after that at 6–12-month intervals
**Lindblom, P., et al. (2001)** [51]	37	8 (21.6)	44	33 (89.2)	NR	NR	NR	NR
**McHenry, C. R. and S. J. Slusarczyk (2000)** [52]	71	25 (35.2)	NR	59 (83.1)	22 (1-86)	TSH > 3.59 μIU/mL	NR	NR
**Meyer, C. D., et al. (2020)** [53]	369	111 (30.1)	43.8 (12.3)	278 (75.3)	74.8 (0.43-160)	NR	NR	NR
**Miller, F. R., et al. (2006)** [54]	90	24 (26.7)	45	73 (81.1)	12.4 (3-24)	TSH > 6.0 mIU/L	6.6	TSH test at least 8 to 10 weeks after surgery and every 3 to 4 months subsequently
**Moon, H. G., et al. (2008)** [55]	101	37 (36.6)	47.5 (12.7)	76 (75.3)	12	NR	NR	TFT at 2 months and every 2 to 3 months during the follow-up period
**Morris, L., et al. (2013)** [56]	98	45 (45.9)	52.5 (13.18)	74 (75.5)	11.6 (1.2-51.3)	NR	7.86	TSH measured 4 to 8 weeks following thyroid lobectomy and then every 6 months during routine follow-up visits
**Ng, P., et al. (2019)** [15]	901	123 (13.7)	45.99 (13.24)	682 (75.7)	65 (3-180)	TSH > 4.5 μIU/mL	21	TSH measured at 6-8 weeks after thyroid surgery, and subsequently at 6 months, 1 year, 2 years, and 5 years or at latest clinic visit
**Noureldine, S. I., et al. (2013)** [5]	105	10 (9.5)	48.4 (11.7)	99 (94.3)	8	TSH > 3.74 μIU/mL	NR	TFT at 4 to 6 weeks
**Park, S., et al. (2017)** [6]	335	215 (64.2)	47.9 (10.5)	266 (79.4)	56.2	TSH > 4.5 mIU/L	3.98	TSH and fT4 measured at 2 to 3 months after lobectomy. Euthyroid patients were monitored every 6 to 12 months thereafter. Once hypothyroidism was diagnosed, all patients were regularly followed up with assessment of thyroid function every 3 to 6 months
**Phitayakorn, R., et al. (2009)** [57]	260	70 (26.9)	48 (15)	217 (83.5)	12 (1-172)	NR	NR	Followed with annual serum TSH level
**Piper, H. G., et al. (2005)** [58]	66	12 (18.2)	46.5	46 (69.7)	NR	TSH > 5.5 μIU/mL	NR	Variable. 70% of patients had an initial TSH drawn within the first 3 months, 12% within 4 to 6 months, 12% within 7 to 12 months, and 6% of patients did not have a TSH drawn within the first year
**Rathi A., S. D., Prasad B. (2017)** [59]	60	19 (31.7)	NR	56 (93.3)	6	NR	2.58	TFT at 1 month, 3 months & 6 months
**Said, M., et al. (2013)** [60]	1240	417 (33.6)	51 (14)	1038 (83.7)	24	TSH > 4 μIU/mL	NR	Median time to the first TSH blood test was 5 weeks
**Salih, A. M. (2018)** [61]	166	1 (0.6)	42.68	139 (83.7)	24	NR	NR	NR
**Sancho, J. J., et al. (2012)** [62]	47	14 (29.8)	41.36 (9.6)	46 (97.9)	55	NR	NR	TSH and fT4 at 3, 6, and 12 months and yearly thereafter
**Sarkis, L. M., et al. (2017)** [63]	276	61 (22.1)	51.5 (15.8)	227 (82.3)	NR	TSH > 4.5 mIU/L	NR	TSH measured 6-8 weeks after surgery
**Seiberling, K. A., et al. (2007)** [64]	58	14 (24.1)	46.5	44 (75.9)	24	TSH > 4 mIU/L	NR	At least one TSH within 6 weeks after surgery
**Sellami, M., et al. (2022)** [65]	214	66 (30.8)	44 (14)	197 (92.1)	NR	TSH > 5 mIU/L	NR	NR
**Spanheimer, P. M., et al. (2011)** [66]	71	24 (33.8)	50.1	NR	7.7	TSH > 4.2 mIU/L	NR	TFTs at 6 weeks
**Stoll, S. J., et al. (2009)** [9]	547	78 (14.3)	50	440 (80.4)	32 (12–54)	TSH > 4.82 mIU/L	NR	A TSH level was measured in all patients approximately 6 to 8 weeks after surgery during routine follow-up
**Su, S. Y., et al. (2009)** [67]	294	32 (10.9)	NR	252 (85.7)	15 (3-150)	TSH > 4 mIU/L	8.2	NR
**Tomoda, C., et al. (2011)** [68]	233	57 (24.5)	51	193 (82.8)	43.2	TSH > 5.0 mIU/l and normal free T4 level lasting for more than 3 months	NR	TSH at least 4–6 weeks after surgery. Serum TSH levels were subsequently followed every 3–6 months for at least 3 years
**Vaiman, M., et al. (2008)** [69]	1051	294 (28)	NR	NR	(24-360)	TSH > 6.0 mIU/L that persisted for at least 8 weeks after surgery	NR	NR
**Wadström, C., et al. (1999)** [70]	229	48 (21)	47	204 (89.1)	168 (12-396)	NR	NR	NR
**Wilson, M., et al. (2020)** [71]	100	47 (47)	50.5 (16.2)	74 (74)	16	NR	NR	TSH at 6 weeks, 6 months, 12 months, and then annually following thyroid lobectomy
**Wormald, R., et al. (2008)** [72]	82	15 (18.3)	53	71 (86.6)	28 (18-38)	TSH > 4.5 mIU/L	5.4 - clinical hypothyroid group 12 - subclinical hypothyroid group	TSH between 3 and 6 months and at 12 months post-operatively. Thereafter, all patients underwent thyroid function testing on an annual basis
**Yetkin, G., et al. (2010)** [73]	104	24 (23)	44.9 (11.9)	92 (88.5)	39.75 (5-87)	TSH > 5 mIU/L	NR	TSH and free thyroxine levels were measured in all patients at the end of the first postoperative month. If the free thyroxine and TSH concentrations were normal, the patients were followed up in 6-week intervals without thyroxine treatment

*NR* Not reported, *TSH* Thyroid stimulating hormone, *TFT* Thyroid function test, *fT4* free thyroxine, *fT3* free triiodothyronine, *T4* Thyroxine, *T3* Triiodothyronine

The duration of follow up varied between 6 months and 168 months, with a median of 25.2 months. There was a variability in terms of the post-operative thyroid function measurement protocols. In some instances, the thyroid function was measured only once at 6 weeks postoperatively, while in others it was measured at 3, 6, and 12 months, with regular 12 monthly tests thereafter. In majority of the studies, hypothyroidism was defined as an elevation in TSH above the upper limit of normal, ranging from 3.74 to 6 μIU/mL.

### Incidence of hypothyroidism

**Fig. 2:**
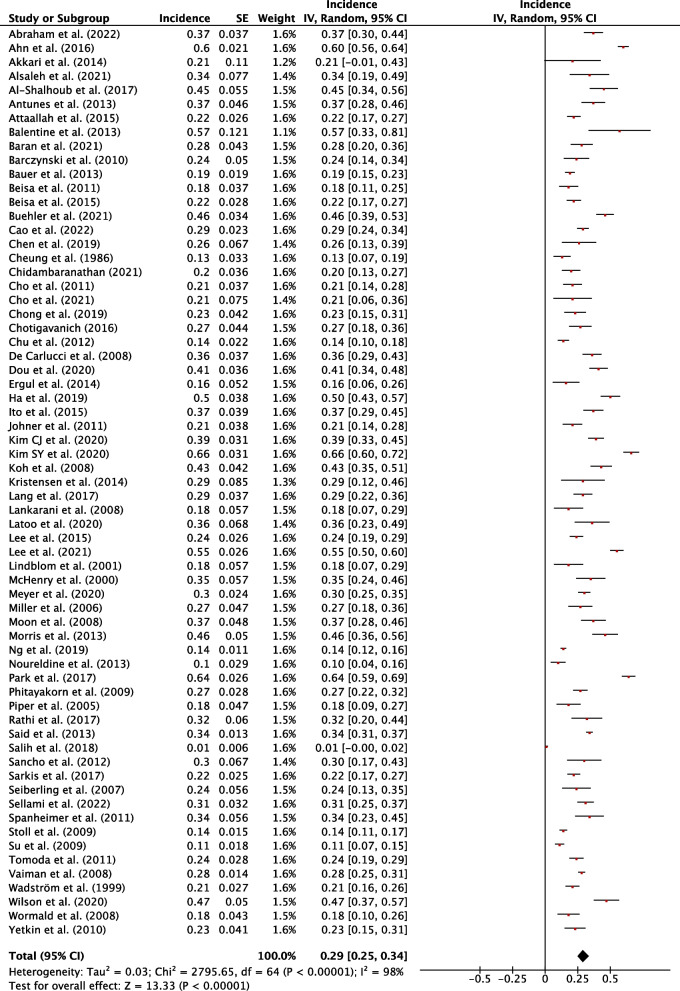
Individual and pooled incidence of hypothyroidism following hemithyroidectomy

**Fig. 3:**
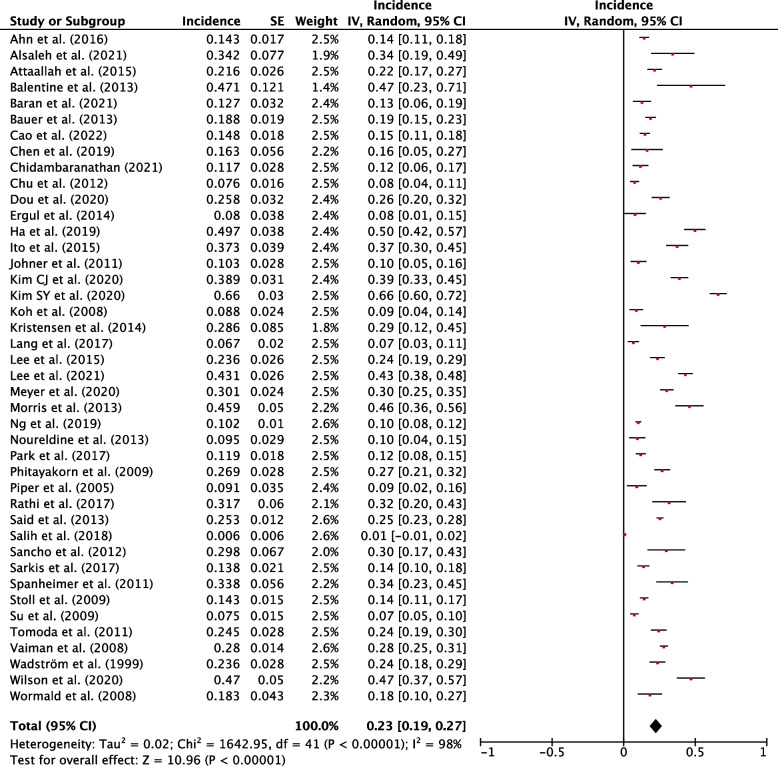
Individual and pooled incidence of thyroxine supplementation following hemithyroidectomy

**Fig. 4:**
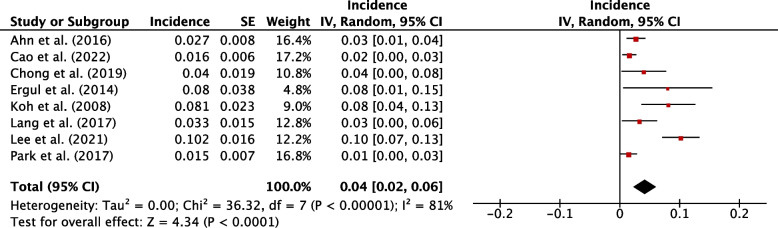
Individual and pooled incidence of overt hypothyroidism following hemithyroidectomy

The weighted pooled incidence of hypothyroidism following hemithyroidectomy was 29% (95% CI, 25-34%; *P*<0.001) (Fig. 2). The weighted pooled incidence of thyroxine supplementation following hemithyroidectomy was 23% (95% CI, 19-27%; *P*<0.001) (Fig. 3). The weighted pooled incidence of overt hypothyroidism was 4% (95% CI, 2-6%, *P*<0.001) (Fig. 4). Each of these three assessments showed a high degree of heterogeneity (1^2 ^> 75%). Funnel plots for these studies qualitatively show asymmetry which may suggest publication bias (Sup. Figures 1-3).

### Risk factors for hypothyroidism

**Fig. 5:**
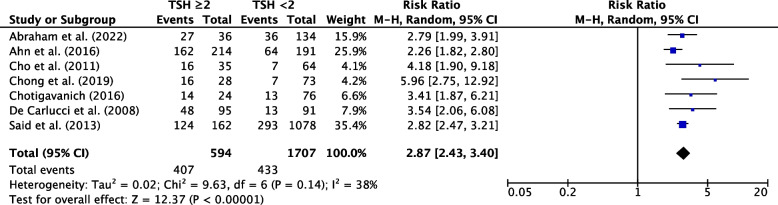
Individual and pooled RR for TSH ≥2 between hypothyroid and euthyroid groups following hemithyroidectomy

Pre-operative TSH was higher in post-operatively hypothyroid patients. The risk ratio for patients with high normal TSH ( ≥ 2 mIU/L) was 2.87 (95% CI, 2.43-3.40; *P*<0.001) (Fig. 5), with a preoperative TSH WMD of 0.87 (95% CI, 0.75-0.98; *P*<0.001) (Sup. Figure 4).

**Fig. 6:**
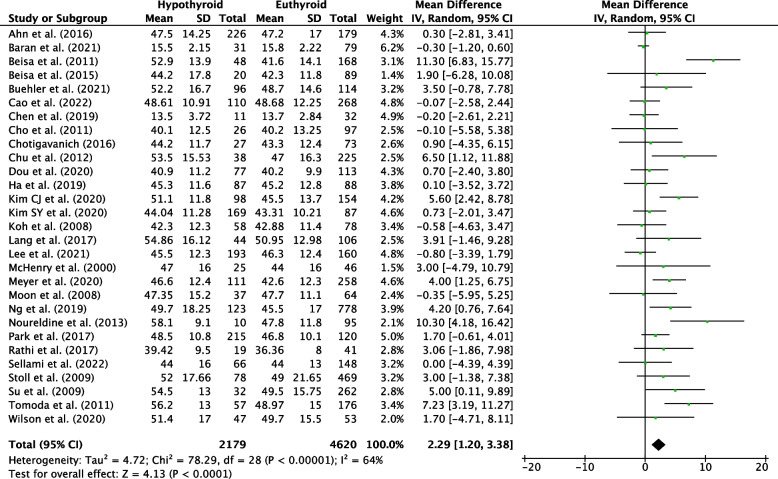
Individual and pooled WMD for age between hypothyroid and euthyroid groups following hemithyroidectomy

Older patients are at greater risk of hypothyroidism. Ages ranged between 39.42-58.1 years in the hypothyroid group in adult studies, and 36.36-50.95 years in euthyroid populations, with a WMD of 2.29 (95% CI, 1.20-3.38; *P*<0.001) (Fig. 6).

**Fig. 7:**
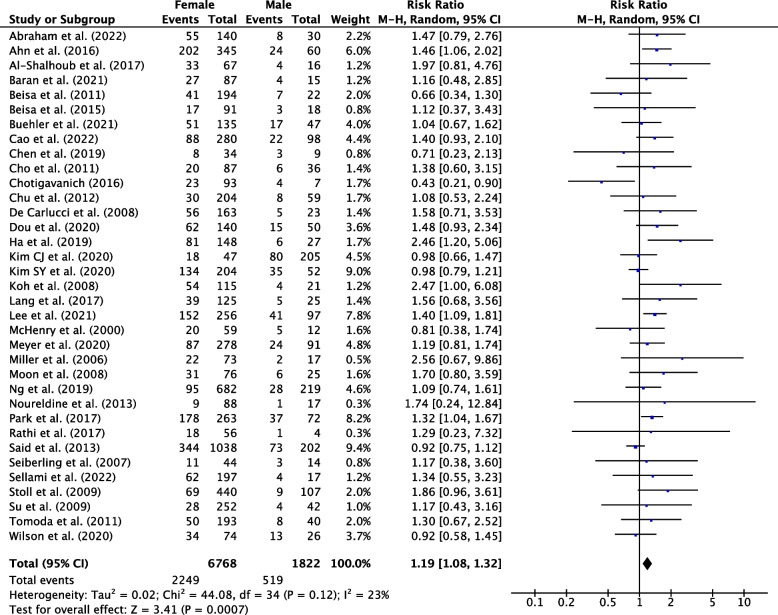
Individual and pooled RR for female sex between hypothyroid and euthyroid groups following hemithyroidectomy

Females were at greater risk of developing hypothyroidism, with a pooled RR of 1.19 (95% CI, 1.08-1.32; *P*=0.007) (Fig. 7).

**Fig. 8:**
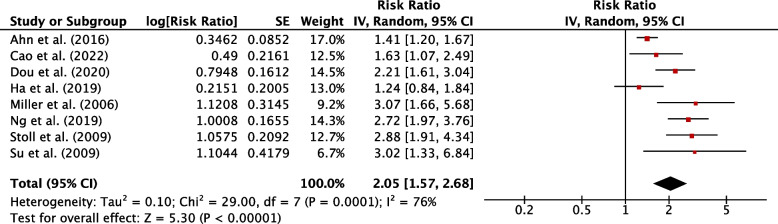
Individual and pooled RR for Hashimoto’s thyroiditis between hypothyroid and euthyroid groups following hemithyroidectomy

Patients with concomitant histopathological Hashimoto’s thyroiditis displayed the greatest single risk in this study for post-hemithyroidectomy hypothyroidism, with a pooled RR of 2.05 (95% CI, 1.57-2.68; *P*=0.001 (Fig. 8). Auto-antibodies are commonly associated with thyroiditis. Both anti-TPO antibody and anti-Tg antibody were associated with an increased risk for hypothyroidism, with a weighted pooled RR of 1.92 (95% CI, 1.49-2.48; *P*<0.001) and 1.53 (95% CI, 1.40-1.88; *P*<0.001) respectively (Sup. Figures 5, 6).

The side of the hemithyroidectomy in this study was also associated with an increased risk of hypothyroidism, with right sided hemithyroidectomies associated with a weighted pooled RR of 1.35 (95% CI, 1.10-1.65, *P*=0.003) (Sup. Figure 7).

A malignant surgical indication, family history of thyroid dysfunction, body mass index (BMI), and remnant thyroid volume were not associated with post-operative hypothyroidism (Sup. Figures 8-11).

Heterogeneity was very high (I^2^ > 75%) in the Hashimoto’s thyroiditis group, moderately high (I^2^ = 50-75%) in the age, TSH, anti-TPO and anti-Tg groups, and low (I^2^ < 50%) for female sex, TSH ≥ 2 mIU/L and side of hemithyroidectomy groups.

Funnel plots for age, female sex, Hashimoto’s thyroiditis, pre-operative TSH, malignant pathology and side of hemithyroidectomy qualitatively show symmetry (Sup. Figures 12-17). Funnel plots for TSH ≥ 2, anti-TPO and anti-Tg qualitatively show asymmetry which may suggest publication bias (Sup. Figures 18-20).

### Post-operative course of hypothyroidism

The average time to hypothyroidism or thyroxine initiation was reported in 19 studies. These values ranged between 1.7-24 months (Table 1). Twelve studies found the average onset of hypothyroidism or thyroxine initiation to be 3-6 months post-operatively.

**Fig. 9:**
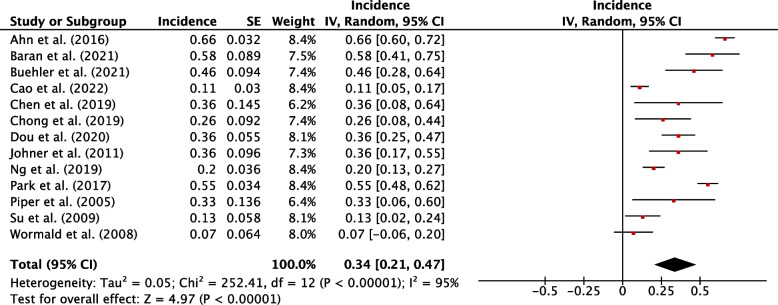
Individual and pooled incidence of transient hypothyroidism following hemithyroidectomy

In these studies 34% (95% CI, 21-47%; *P*<0.001) of postoperatively hypothyroid patients recovered to euthyroid status (Fig. 9), displaying transient hypothyroidism. Four papers assessed the factors associated with transient hypothyroidism [6–8, 13] (Sup. Table 2). Three studies found patients with a lower pre-operative TSH had a higher likelihood of returning to euthyroidism [6–8]. A funnel plot assessing transient hypothyroidism qualitatively showed asymmetry which may indicate publication bias (Sup. Figure 21).

## Discussion

The incidence of hypothyroidism following hemithyroidectomy was 29%, and the incidence of thyroxine supplementation alone 23%. The higher rate for hypothyroidism reflects that many studies did not initiate thyroxine for biochemical hypothyroidism alone but typically for clinical or overt hypothyroidism or TSH significantly above the upper limit of normal. The incidence of overt hypothyroidism was 4% which may be of significance for the clinician and patient due to its health effects.

A high degree of heterogeneity was recorded in the pooled incidence of hypothyroidism, thyroxine supplementation, and overt or clinical hypothyroidism. This may be explained by the differences in study follow up protocol, definitions for hypothyroidism, or indications for thyroxine supplementation. The first thyroid function (TF) measurements occurred at varying times, ranging from 1 week to 3-6 months post-surgery. Further, follow up protocol varied by duration of follow up, as well as frequency of thyroid function measurements; the follow up duration ranged from 6 to 168 months, and thyroid function measurements ranged from occurring only once, to several times at regular intervals. Studies typically defined hypothyroidism as an increase in TSH above the upper limit of normal, however the accepted upper limit of normal at each institution ranged from 3.74 to 6 μIU/mL^5^. Greater frequency of TF measurements, and greater duration of follow up, as well as a more liberal definition of hypothyroidism will likely increase the detection of hypothyroidism. Further, the surgical definition of hemithyroidectomy varied, with some studies characterising the procedure by lobectomy plus isthmusectomy, and others by lobectomy alone [13]. Resection of the isthmus may reduce the total thyroid volume and reduce the post-operative thyroid function, further adding to the variation. Finally, some studies administered thyroxine for any elevation of TSH above the normal range while others only for clinically symptomatic or overtly hypothyroid patients, which may contribute to the high degree of heterogeneity.

The risk factors identified for the development of hypothyroidism included higher pre-operative TSH, older age, female sex, Hashimoto’s thyroiditis, thyroid autoantibodies, and right sided hemithyroidectomy.

Higher pre-operative TSH levels are regularly cited as a risk factor for hypothyroidism. A higher TSH level indicates that the thyroid may already be dysfunctional prior to surgery, and more vulnerable to hypothyroidism. Older age was also found to be significantly associated. Age has been observed to be associated with thyroid decline and elevated TSH levels [76–78], with studies showing a TSH increase of 0.08 mU/L per decade of age [79]which may explain the preponderance of older patients in the hypothyroid group. Females were at increased risk of hypothyroidism which may be due to a higher rate of concomitant thyroid disorders amongst women [11], such as Hashimoto’s which has been reported amongst women at a rate five times men [78].

Auto-antibodies are associated with thyroiditis and play a role in the auto-immune mediated dysfunction of the thyroid gland [80], likely explaining the association of auto-autoantibodies with hypothyroidism. The side of hemithyroidectomy may also significantly increase the risk of hypothyroidism. Hashimoto’s thyroiditis is the most common subset of thyroiditis and is characterised by auto-immune destruction and progressive fibrosis of the thyroid gland [79], leading to thyroid dysfunction. Some studies have shown thyroid lobe asymmetry with the right lobe larger than the left [81, 82], possibly explaining the higher incidence of hypothyroidism in right sided hemithyroidectomy.

This study was novel in its analysis of post-operative thyroid function course, with the majority of studies finding the average onset of hypothyroidism or thyroxine initiation to be 3-6 months post-operatively. In a practical sense this can raise the awareness of early postoperative hypothyroidism and guide patient follow up. The pooled incidence of transient hypothyroidism representing spontaneous recovery of thyroid function without thyroxine supplementation was 34%. This may reflect gradual thyroid compensation. Seven studies reported average post-operative TSH levels at multiple time points post-operatively [8, 12, 25, 27, 45, 60, 68]. In six studies, the post-operative TSH peaked within 6 months, before declining over the subsequent months, in one study returning from a mean of 4.21 to 2.85 μIU/mL between 3 and 12 months postoperatively [12]. This natural recovery of thyroid function and decline in TSH level may explain the incidence of transient hypothyroidism. This indicates the need for regular follow up and monitoring of thyroid function. Transient hypothyroidism may also affect the interpretation of hypothyroid risk, with the incidence potentially higher than the prevalence.

This study is limited in a couple of respects. Firstly, studies reported hypothyroidism or initiated thyroxine supplementation according to a wide range of definitions or indications. Second studies followed institution dependant follow up protocols which varied significantly by their duration and the frequency of measurement. This lack of consistency across studies will reduce the applicability of our findings. Finally, the administration of levothyroxine in hypothyroid patients may mask a recovery in thyroid function, which may affect the observed rate of transient hypothyroidism.

This systematic review and meta-analysis is useful for clinicians in advising patients on post-operative risks and choosing the most suitable intervention for the patient. Those at high risk for hypothyroidism may elect to defer surgery if this is open to them or choose to undergo a total thyroidectomy to limit recurrence of the pathology being treated. Further, awareness of the post-surgical hypothyroid time course will inform follow up regimens and improve awareness of transient hypothyroidism which may reduce unnecessary treatment of biochemical hypothyroidism.

## Conclusion

This systematic review and meta-analysis found the incidence of hypothyroidism post-hemithyroidectomy to be high suggesting the needs for better treatment strategy including more careful surgical approach by the surgeon to reduce this associated complication of hemithyroidectomy among the patients at risk. Higher pre-operative TSH, older age, female sex, Hashimoto’s thyroiditis, autoantibodies anti-TPO and anti-Tg and right sided hemithyroidectomy were found to increase the risk of hypothyroidism post-operatively. Patients should be individually risk assessed for the development of hypothyroidism and counselled accordingly. Further research is required to explore the factors associated with transient hypothyroidism and the length of time required for recovery and to identify the factors associated with late onset hypothyroidism.

### Supplementary Information

Supplementary Material 1: Supplementary Figure 1. Funnel plot for incidence of hypothyroidism following hemithyroidectomy. Supplementary Figure 2. Funnel plot for incidence of thyroxine supplementation following hemithyroidectomy. Supplementary Figure 3. Funnel plot for incidence of overt hypothyroidism following hemithyroidectomy. Supplementary Figure 4. Individual and pooled WMD for pre-operative TSH between hypothyroid and euthyroid groups following hemithyroidectomy. Supplementary Figure 5. Individual and pooled RR for pre-operative anti-TPO positivity between hypothyroid and euthyroid groups following hemithyroidectomy. Supplementary Figure 6. Individual and pooled RR for pre-operative anti-Tg positivity between hypothyroid and euthyroid groups following hemithyroidectomy. Supplementary Figure 7. Individual and pooled RR for right sided hemithyroidectomy between hypothyroid and euthyroid groups following hemithyroidectomy. Supplementary Figure 8. Individual and pooled RR for malignant pathology between hypothyroid and euthyroid groups following hemithyroidectomy. Supplementary Figure 9. Individual and pooled RR of postoperative hypothyroidism for patients with a family of thyroid dysfunction.Supplementary Figure 10. Individual and pooled WMD of BMI between hypothyroid and euthyroid groups. Supplementary Figure 11. Individual and pooled WMD of remnant thyroid volume between hypothyroid and euthyroid groups. Supplementary Figure 12. Funnel plot assessing asymmetry of WMD for age between hypothyroid and euthyroid groups. Supplementary Figure 13. Funnel plot assessing asymmetry of RR of female sex between hypothyroid and euthyroid groups. Supplementary Figure 14. Funnel plot assessing asymmetry of RR for Hashimoto’s thyroiditis between hypothyroid and euthyroid groups. Supplementary Figure 15. Funnel plot assessing asymmetry of WMD of pre-operative TSH between hypothyroid and euthyroid groups. Supplementary Figure 16. Funnel plot assessing asymmetry of RR for malignant pathology between hypothyroid and euthyroid groups. Supplementary Figure 17. Funnel plot assessing asymmetry of RR for side of hemithyroidectomy between hypothyroid and euthyroid groups. Supplementary Figure 18. Funnel plot assessing asymmetry of RR for TSH ≥2 between hypothyroid and euthyroid groups. Supplementary Figure 19. Funnel plot assessing asymmetry of RR for anti-TPO between hypothyroid and euthyroid groups. Supplementary Figure 20. Funnel plot assessing asymmetry of RR for anti-Tg between hypothyroid and euthyroid groups. Supplementary Figure 21. Funnel plot assessing asymmetry for incidence of transient hypothyroidism following hemithyroidectomy.

Supplementary Material 2.

## Data Availability

No datasets were generated or analysed during the current study.
