# Supplementary figures and images for: Hypothyroidism after hemithyroidectomy: a systematic review and meta-analysis
Source: Thyroid Res. 2024 Jul 8;17:18. doi: 10.1186/s13044-024-00200-z (PMC11229296; doi:10.1186/s13044-024-00200-z)

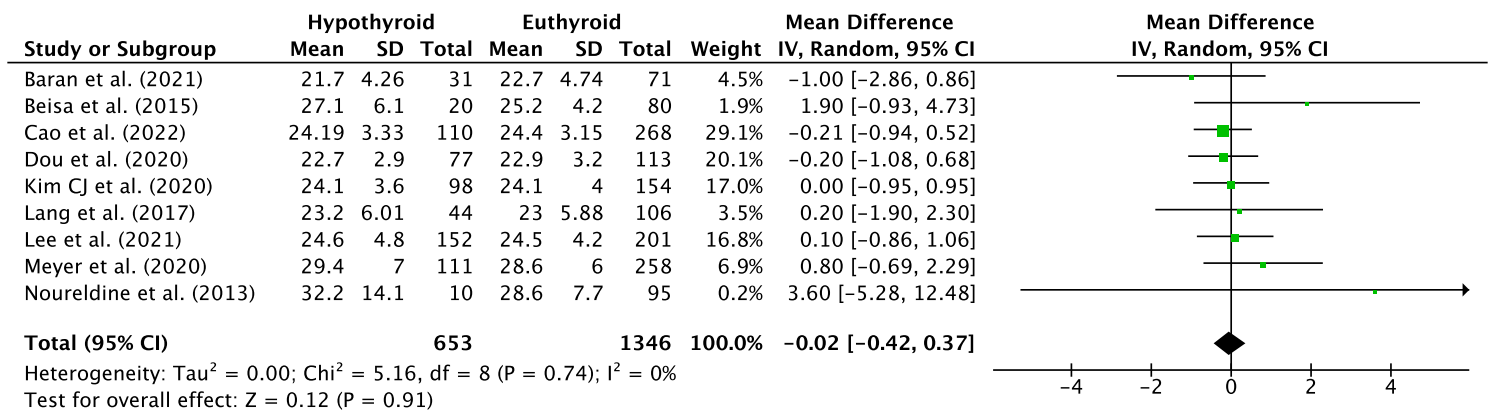

Supplement: Supplementary file 1 — Supplementary Material 1: Supplementary Figure 1. Funnel plot for incidence of hypothyroidism following hemithyroidectomy. Supplementary Figure 2. Funnel plot for incidence of thyroxine supplementation following hemithyroidectomy. Supplementary Figure 3. Funnel plot for incidence of overt hypothyroidism following hemithyroidectomy. Supplementary Figure 4. Individual and pooled WMD for pre-operative TSH between hypothyroid and euthyroid groups following hemithyroidectomy. Supplementary Figure 5. Individual and pooled RR for pre-operative anti-TPO positivity between hypothyroid and euthyroid groups following hemithyroidectomy. Supplementary Figure 6. Individual and pooled RR for pre-operative anti-Tg positivity between hypothyroid and euthyroid groups following hemithyroidectomy. Supplementary Figure 7. Individual and pooled RR for right sided hemithyroidectomy between hypothyroid and euthyroid groups following hemithyroidectomy. Supplementary Figure 8. Individual and pooled RR for malignant pathology between hypothyroid and euthyroid groups following hemithyroidectomy. Supplementary Figure 9. Individual and pooled RR of postoperative hypothyroidism for patients with a family of thyroid dysfunction.Supplementary Figure 10. Individual and pooled WMD of BMI between hypothyroid and euthyroid groups. Supplementary Figure 11. Individual and pooled WMD of remnant thyroid volume between hypothyroid and euthyroid groups. Supplementary Figure 12. Funnel plot assessing asymmetry of WMD for age between hypothyroid and euthyroid groups. Supplementary Figure 13. Funnel plot assessing asymmetry of RR of female sex between hypothyroid and euthyroid groups. Supplementary Figure 14. Funnel plot assessing asymmetry of RR for Hashimoto’s thyroiditis between hypothyroid and euthyroid groups. Supplementary Figure 15. Funnel plot assessing asymmetry of WMD of pre-operative TSH between hypothyroid and euthyroid groups. Supplementary Figure 16. Funnel plot assessing asymmetry of RR for malig [file 13044_2024_200_MOESM1_ESM.zip › SF10BMI.pdf]

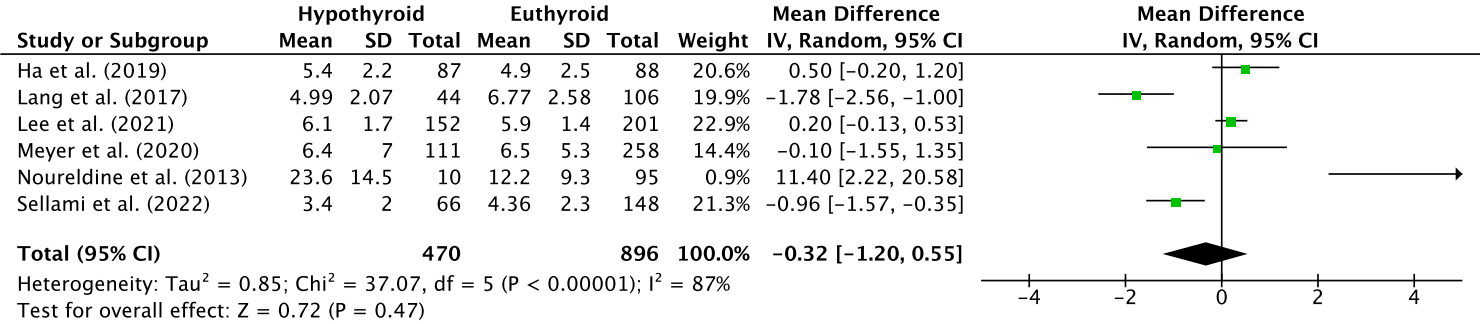

Supplement: Supplementary file 1 — Supplementary Material 1: Supplementary Figure 1. Funnel plot for incidence of hypothyroidism following hemithyroidectomy. Supplementary Figure 2. Funnel plot for incidence of thyroxine supplementation following hemithyroidectomy. Supplementary Figure 3. Funnel plot for incidence of overt hypothyroidism following hemithyroidectomy. Supplementary Figure 4. Individual and pooled WMD for pre-operative TSH between hypothyroid and euthyroid groups following hemithyroidectomy. Supplementary Figure 5. Individual and pooled RR for pre-operative anti-TPO positivity between hypothyroid and euthyroid groups following hemithyroidectomy. Supplementary Figure 6. Individual and pooled RR for pre-operative anti-Tg positivity between hypothyroid and euthyroid groups following hemithyroidectomy. Supplementary Figure 7. Individual and pooled RR for right sided hemithyroidectomy between hypothyroid and euthyroid groups following hemithyroidectomy. Supplementary Figure 8. Individual and pooled RR for malignant pathology between hypothyroid and euthyroid groups following hemithyroidectomy. Supplementary Figure 9. Individual and pooled RR of postoperative hypothyroidism for patients with a family of thyroid dysfunction.Supplementary Figure 10. Individual and pooled WMD of BMI between hypothyroid and euthyroid groups. Supplementary Figure 11. Individual and pooled WMD of remnant thyroid volume between hypothyroid and euthyroid groups. Supplementary Figure 12. Funnel plot assessing asymmetry of WMD for age between hypothyroid and euthyroid groups. Supplementary Figure 13. Funnel plot assessing asymmetry of RR of female sex between hypothyroid and euthyroid groups. Supplementary Figure 14. Funnel plot assessing asymmetry of RR for Hashimoto’s thyroiditis between hypothyroid and euthyroid groups. Supplementary Figure 15. Funnel plot assessing asymmetry of WMD of pre-operative TSH between hypothyroid and euthyroid groups. Supplementary Figure 16. Funnel plot assessing asymmetry of RR for malig [file 13044_2024_200_MOESM1_ESM.zip › SF11RemnantVolume.pdf]

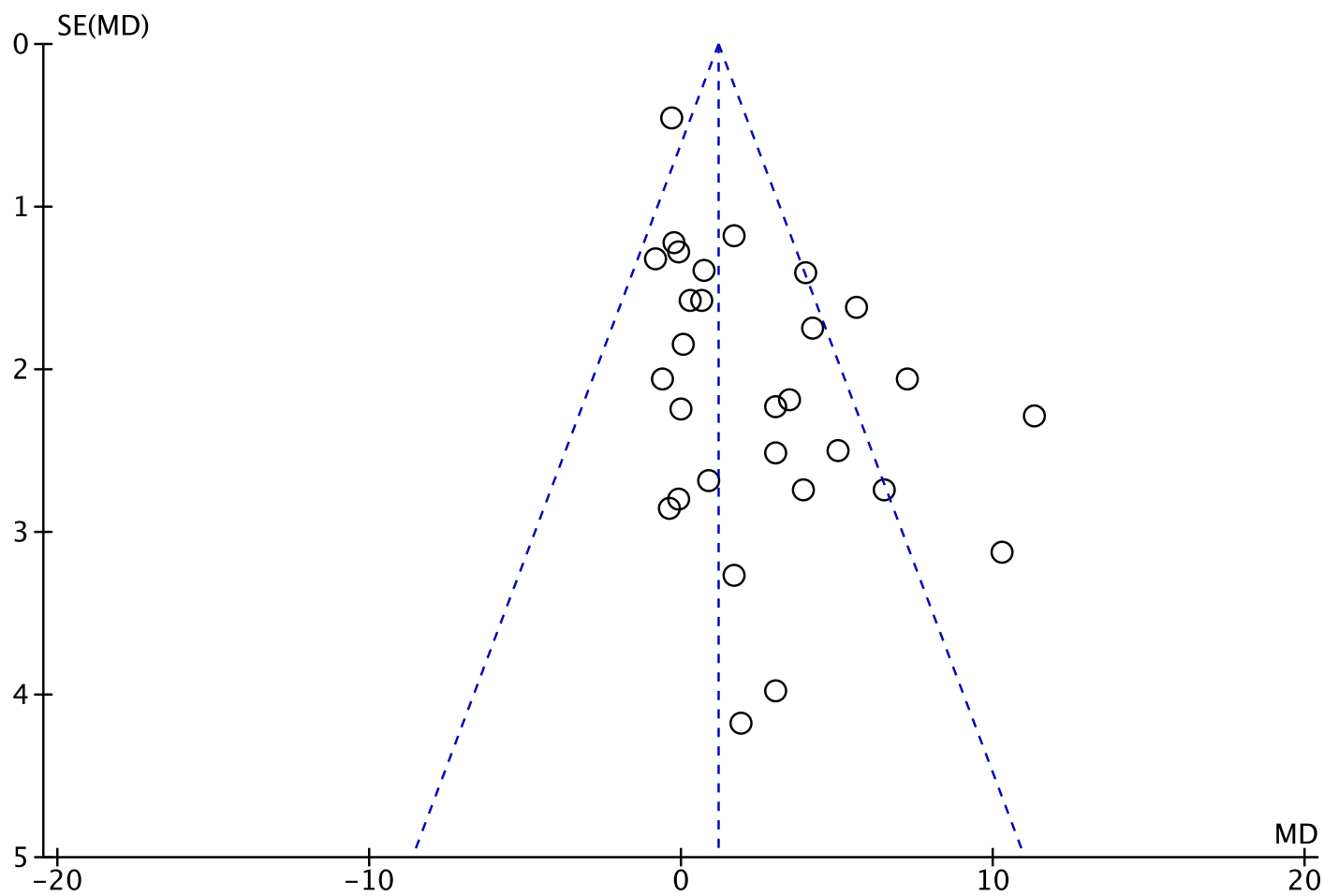

Supplement: Supplementary file 1 — Supplementary Material 1: Supplementary Figure 1. Funnel plot for incidence of hypothyroidism following hemithyroidectomy. Supplementary Figure 2. Funnel plot for incidence of thyroxine supplementation following hemithyroidectomy. Supplementary Figure 3. Funnel plot for incidence of overt hypothyroidism following hemithyroidectomy. Supplementary Figure 4. Individual and pooled WMD for pre-operative TSH between hypothyroid and euthyroid groups following hemithyroidectomy. Supplementary Figure 5. Individual and pooled RR for pre-operative anti-TPO positivity between hypothyroid and euthyroid groups following hemithyroidectomy. Supplementary Figure 6. Individual and pooled RR for pre-operative anti-Tg positivity between hypothyroid and euthyroid groups following hemithyroidectomy. Supplementary Figure 7. Individual and pooled RR for right sided hemithyroidectomy between hypothyroid and euthyroid groups following hemithyroidectomy. Supplementary Figure 8. Individual and pooled RR for malignant pathology between hypothyroid and euthyroid groups following hemithyroidectomy. Supplementary Figure 9. Individual and pooled RR of postoperative hypothyroidism for patients with a family of thyroid dysfunction.Supplementary Figure 10. Individual and pooled WMD of BMI between hypothyroid and euthyroid groups. Supplementary Figure 11. Individual and pooled WMD of remnant thyroid volume between hypothyroid and euthyroid groups. Supplementary Figure 12. Funnel plot assessing asymmetry of WMD for age between hypothyroid and euthyroid groups. Supplementary Figure 13. Funnel plot assessing asymmetry of RR of female sex between hypothyroid and euthyroid groups. Supplementary Figure 14. Funnel plot assessing asymmetry of RR for Hashimoto’s thyroiditis between hypothyroid and euthyroid groups. Supplementary Figure 15. Funnel plot assessing asymmetry of WMD of pre-operative TSH between hypothyroid and euthyroid groups. Supplementary Figure 16. Funnel plot assessing asymmetry of RR for malig [file 13044_2024_200_MOESM1_ESM.zip › SF12AgeFunnel.pdf]

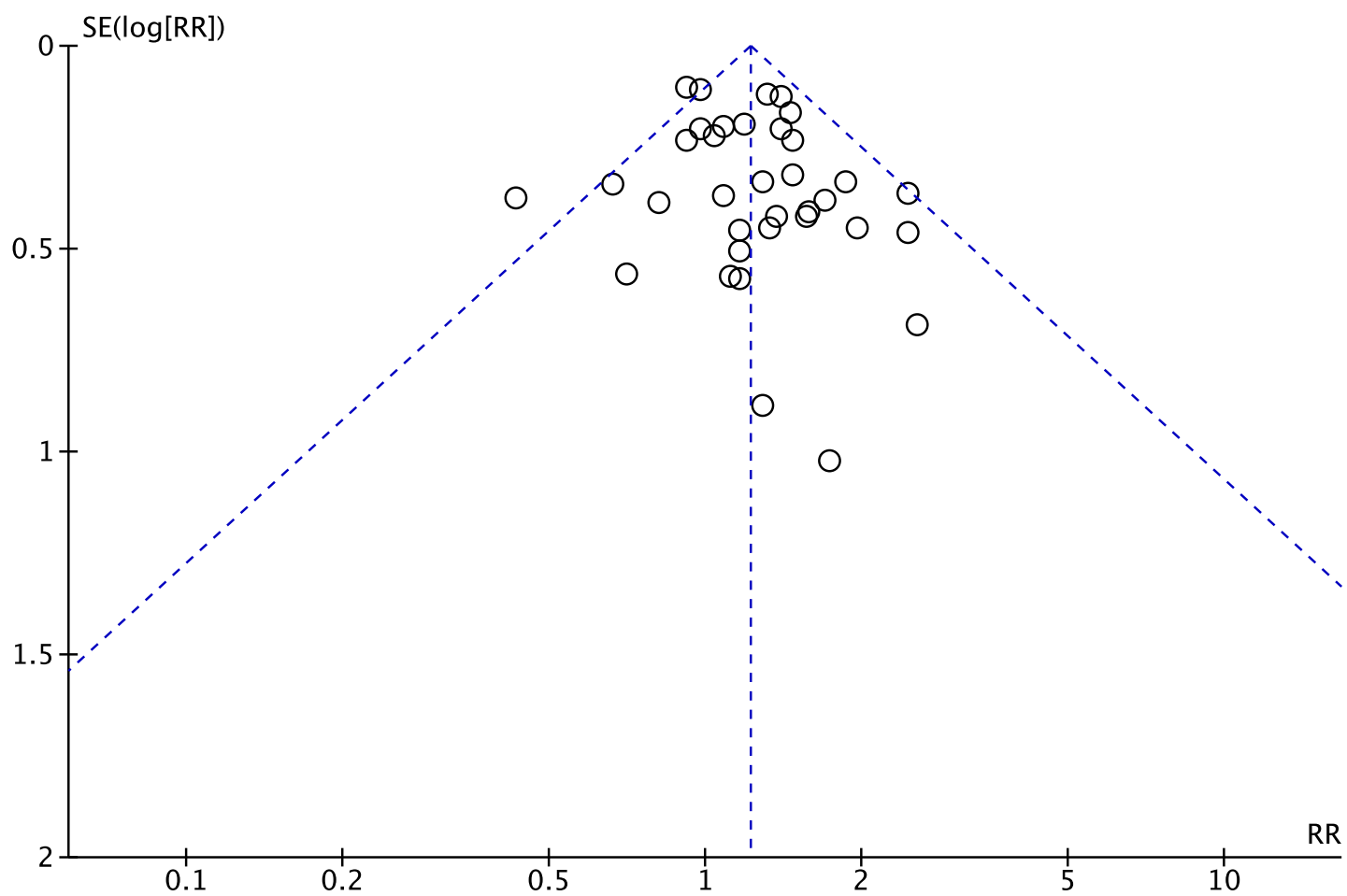

Supplement: Supplementary file 1 — Supplementary Material 1: Supplementary Figure 1. Funnel plot for incidence of hypothyroidism following hemithyroidectomy. Supplementary Figure 2. Funnel plot for incidence of thyroxine supplementation following hemithyroidectomy. Supplementary Figure 3. Funnel plot for incidence of overt hypothyroidism following hemithyroidectomy. Supplementary Figure 4. Individual and pooled WMD for pre-operative TSH between hypothyroid and euthyroid groups following hemithyroidectomy. Supplementary Figure 5. Individual and pooled RR for pre-operative anti-TPO positivity between hypothyroid and euthyroid groups following hemithyroidectomy. Supplementary Figure 6. Individual and pooled RR for pre-operative anti-Tg positivity between hypothyroid and euthyroid groups following hemithyroidectomy. Supplementary Figure 7. Individual and pooled RR for right sided hemithyroidectomy between hypothyroid and euthyroid groups following hemithyroidectomy. Supplementary Figure 8. Individual and pooled RR for malignant pathology between hypothyroid and euthyroid groups following hemithyroidectomy. Supplementary Figure 9. Individual and pooled RR of postoperative hypothyroidism for patients with a family of thyroid dysfunction.Supplementary Figure 10. Individual and pooled WMD of BMI between hypothyroid and euthyroid groups. Supplementary Figure 11. Individual and pooled WMD of remnant thyroid volume between hypothyroid and euthyroid groups. Supplementary Figure 12. Funnel plot assessing asymmetry of WMD for age between hypothyroid and euthyroid groups. Supplementary Figure 13. Funnel plot assessing asymmetry of RR of female sex between hypothyroid and euthyroid groups. Supplementary Figure 14. Funnel plot assessing asymmetry of RR for Hashimoto’s thyroiditis between hypothyroid and euthyroid groups. Supplementary Figure 15. Funnel plot assessing asymmetry of WMD of pre-operative TSH between hypothyroid and euthyroid groups. Supplementary Figure 16. Funnel plot assessing asymmetry of RR for malig [file 13044_2024_200_MOESM1_ESM.zip › SF13FemaleSexFunnel.pdf]

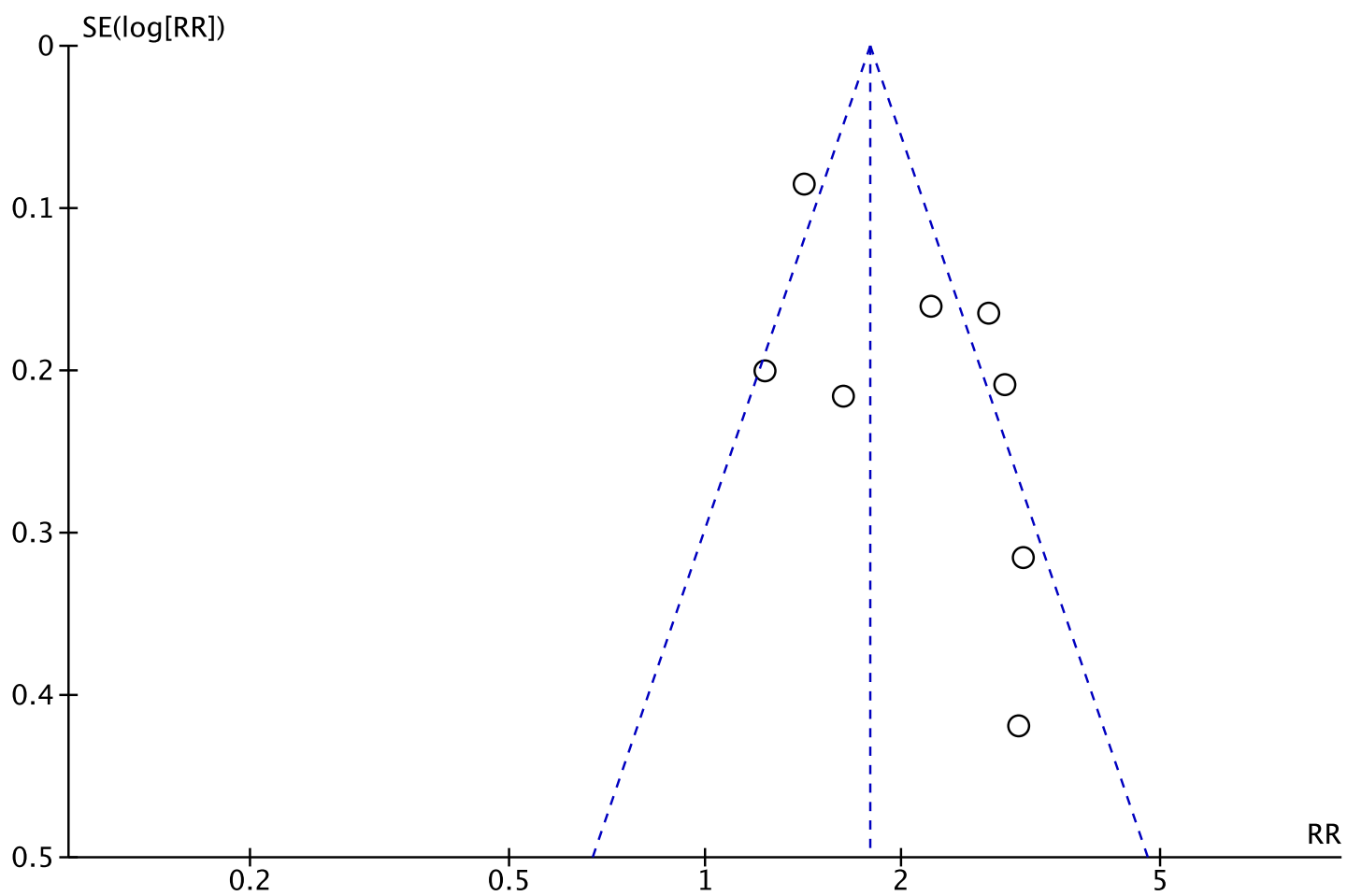

Supplement: Supplementary file 1 — Supplementary Material 1: Supplementary Figure 1. Funnel plot for incidence of hypothyroidism following hemithyroidectomy. Supplementary Figure 2. Funnel plot for incidence of thyroxine supplementation following hemithyroidectomy. Supplementary Figure 3. Funnel plot for incidence of overt hypothyroidism following hemithyroidectomy. Supplementary Figure 4. Individual and pooled WMD for pre-operative TSH between hypothyroid and euthyroid groups following hemithyroidectomy. Supplementary Figure 5. Individual and pooled RR for pre-operative anti-TPO positivity between hypothyroid and euthyroid groups following hemithyroidectomy. Supplementary Figure 6. Individual and pooled RR for pre-operative anti-Tg positivity between hypothyroid and euthyroid groups following hemithyroidectomy. Supplementary Figure 7. Individual and pooled RR for right sided hemithyroidectomy between hypothyroid and euthyroid groups following hemithyroidectomy. Supplementary Figure 8. Individual and pooled RR for malignant pathology between hypothyroid and euthyroid groups following hemithyroidectomy. Supplementary Figure 9. Individual and pooled RR of postoperative hypothyroidism for patients with a family of thyroid dysfunction.Supplementary Figure 10. Individual and pooled WMD of BMI between hypothyroid and euthyroid groups. Supplementary Figure 11. Individual and pooled WMD of remnant thyroid volume between hypothyroid and euthyroid groups. Supplementary Figure 12. Funnel plot assessing asymmetry of WMD for age between hypothyroid and euthyroid groups. Supplementary Figure 13. Funnel plot assessing asymmetry of RR of female sex between hypothyroid and euthyroid groups. Supplementary Figure 14. Funnel plot assessing asymmetry of RR for Hashimoto’s thyroiditis between hypothyroid and euthyroid groups. Supplementary Figure 15. Funnel plot assessing asymmetry of WMD of pre-operative TSH between hypothyroid and euthyroid groups. Supplementary Figure 16. Funnel plot assessing asymmetry of RR for malig [file 13044_2024_200_MOESM1_ESM.zip › SF14HashimotoFunnel.pdf]

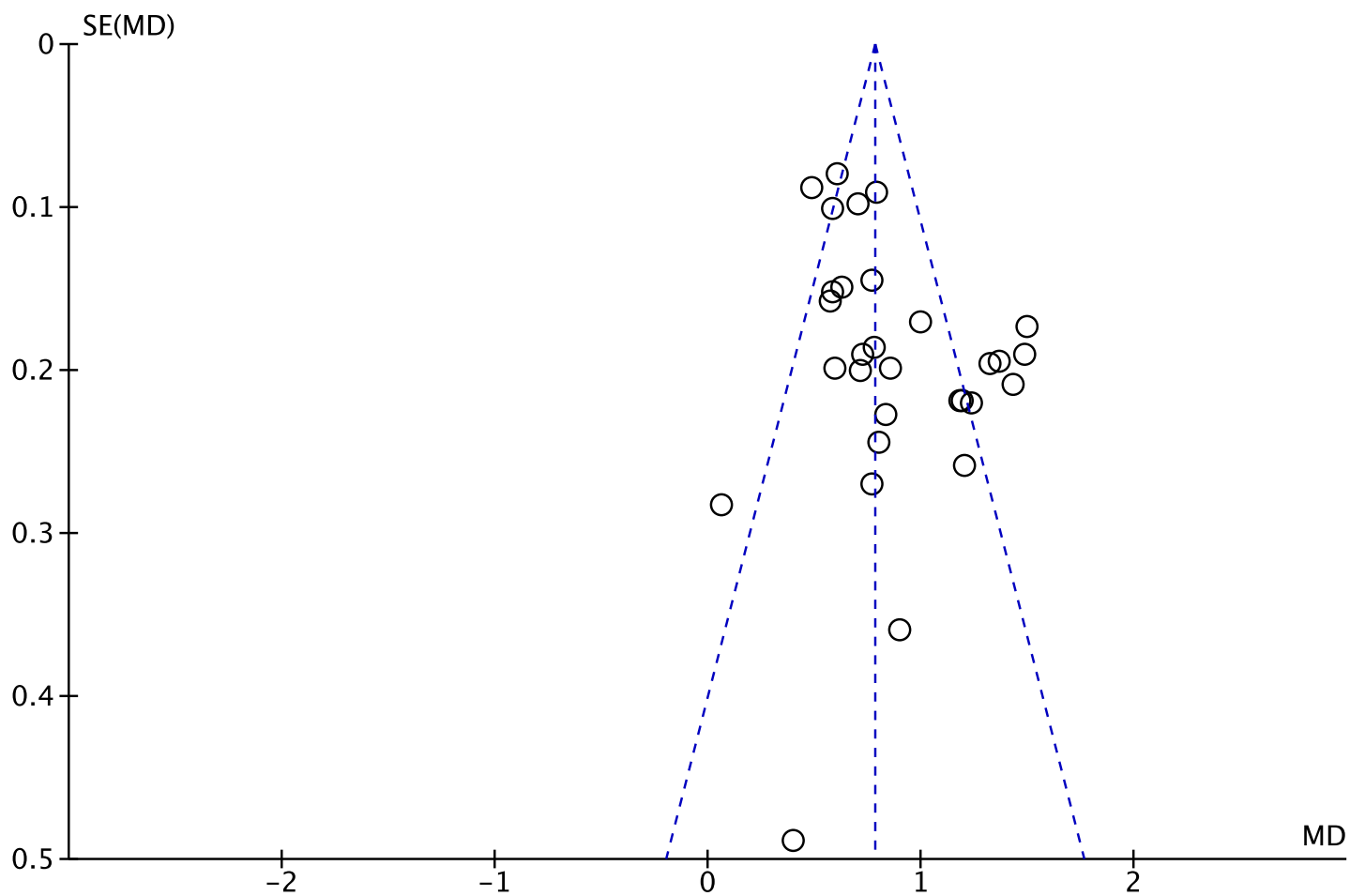

Supplement: Supplementary file 1 — Supplementary Material 1: Supplementary Figure 1. Funnel plot for incidence of hypothyroidism following hemithyroidectomy. Supplementary Figure 2. Funnel plot for incidence of thyroxine supplementation following hemithyroidectomy. Supplementary Figure 3. Funnel plot for incidence of overt hypothyroidism following hemithyroidectomy. Supplementary Figure 4. Individual and pooled WMD for pre-operative TSH between hypothyroid and euthyroid groups following hemithyroidectomy. Supplementary Figure 5. Individual and pooled RR for pre-operative anti-TPO positivity between hypothyroid and euthyroid groups following hemithyroidectomy. Supplementary Figure 6. Individual and pooled RR for pre-operative anti-Tg positivity between hypothyroid and euthyroid groups following hemithyroidectomy. Supplementary Figure 7. Individual and pooled RR for right sided hemithyroidectomy between hypothyroid and euthyroid groups following hemithyroidectomy. Supplementary Figure 8. Individual and pooled RR for malignant pathology between hypothyroid and euthyroid groups following hemithyroidectomy. Supplementary Figure 9. Individual and pooled RR of postoperative hypothyroidism for patients with a family of thyroid dysfunction.Supplementary Figure 10. Individual and pooled WMD of BMI between hypothyroid and euthyroid groups. Supplementary Figure 11. Individual and pooled WMD of remnant thyroid volume between hypothyroid and euthyroid groups. Supplementary Figure 12. Funnel plot assessing asymmetry of WMD for age between hypothyroid and euthyroid groups. Supplementary Figure 13. Funnel plot assessing asymmetry of RR of female sex between hypothyroid and euthyroid groups. Supplementary Figure 14. Funnel plot assessing asymmetry of RR for Hashimoto’s thyroiditis between hypothyroid and euthyroid groups. Supplementary Figure 15. Funnel plot assessing asymmetry of WMD of pre-operative TSH between hypothyroid and euthyroid groups. Supplementary Figure 16. Funnel plot assessing asymmetry of RR for malig [file 13044_2024_200_MOESM1_ESM.zip › SF15TSHFunnel.pdf]

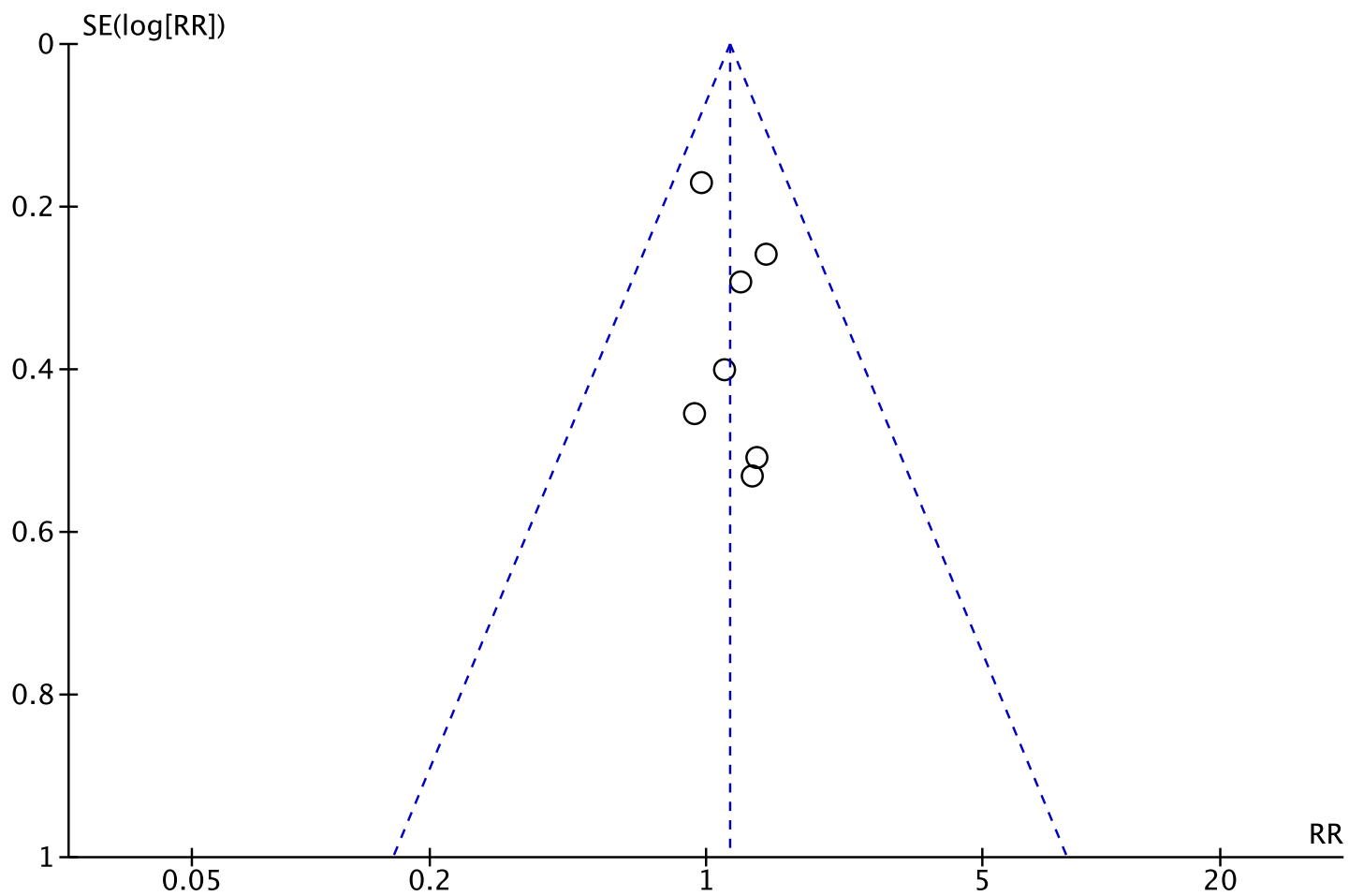

Supplement: Supplementary file 1 — Supplementary Material 1: Supplementary Figure 1. Funnel plot for incidence of hypothyroidism following hemithyroidectomy. Supplementary Figure 2. Funnel plot for incidence of thyroxine supplementation following hemithyroidectomy. Supplementary Figure 3. Funnel plot for incidence of overt hypothyroidism following hemithyroidectomy. Supplementary Figure 4. Individual and pooled WMD for pre-operative TSH between hypothyroid and euthyroid groups following hemithyroidectomy. Supplementary Figure 5. Individual and pooled RR for pre-operative anti-TPO positivity between hypothyroid and euthyroid groups following hemithyroidectomy. Supplementary Figure 6. Individual and pooled RR for pre-operative anti-Tg positivity between hypothyroid and euthyroid groups following hemithyroidectomy. Supplementary Figure 7. Individual and pooled RR for right sided hemithyroidectomy between hypothyroid and euthyroid groups following hemithyroidectomy. Supplementary Figure 8. Individual and pooled RR for malignant pathology between hypothyroid and euthyroid groups following hemithyroidectomy. Supplementary Figure 9. Individual and pooled RR of postoperative hypothyroidism for patients with a family of thyroid dysfunction.Supplementary Figure 10. Individual and pooled WMD of BMI between hypothyroid and euthyroid groups. Supplementary Figure 11. Individual and pooled WMD of remnant thyroid volume between hypothyroid and euthyroid groups. Supplementary Figure 12. Funnel plot assessing asymmetry of WMD for age between hypothyroid and euthyroid groups. Supplementary Figure 13. Funnel plot assessing asymmetry of RR of female sex between hypothyroid and euthyroid groups. Supplementary Figure 14. Funnel plot assessing asymmetry of RR for Hashimoto’s thyroiditis between hypothyroid and euthyroid groups. Supplementary Figure 15. Funnel plot assessing asymmetry of WMD of pre-operative TSH between hypothyroid and euthyroid groups. Supplementary Figure 16. Funnel plot assessing asymmetry of RR for malig [file 13044_2024_200_MOESM1_ESM.zip › SF16MalignantFunnel.pdf]

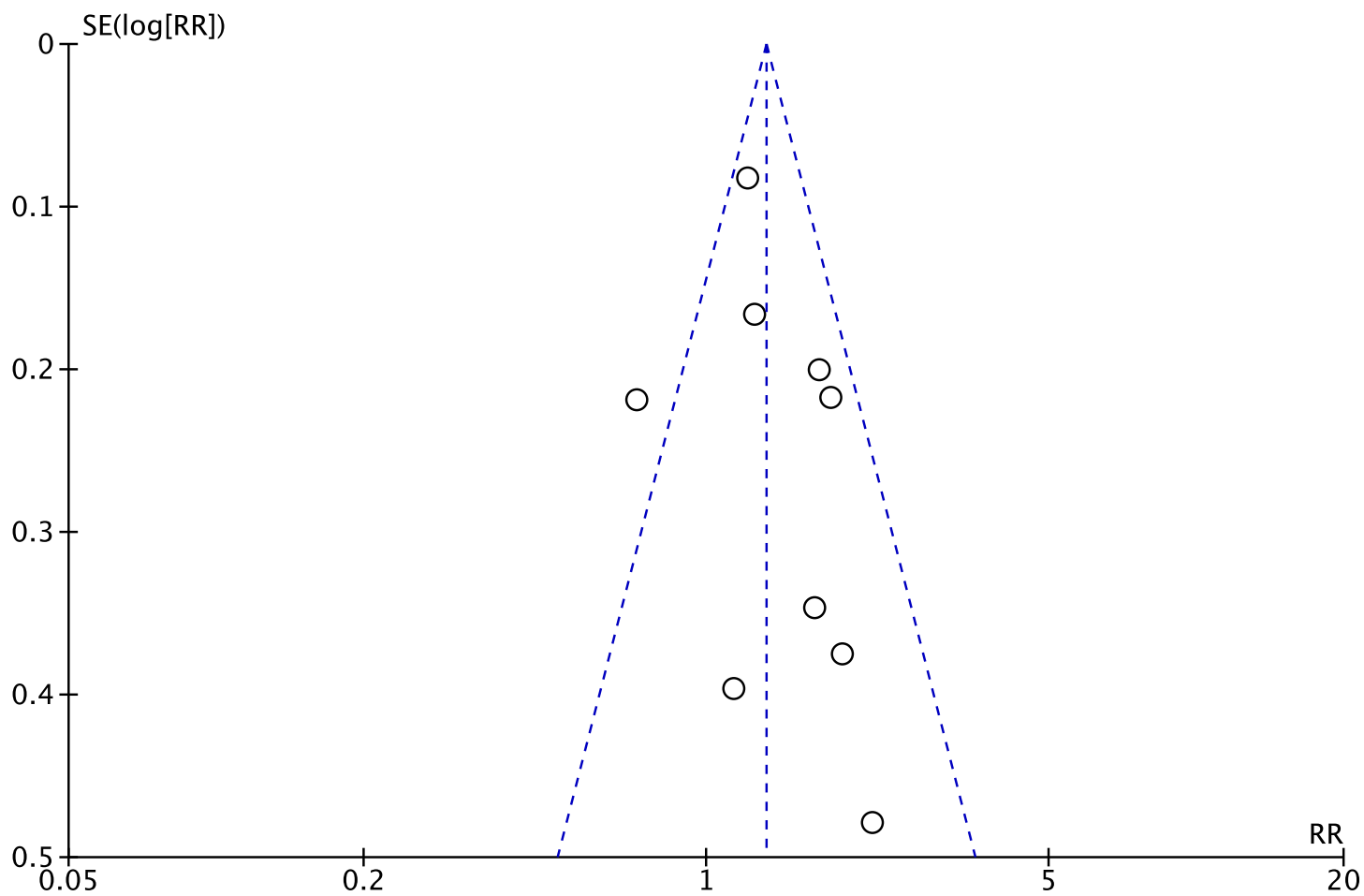

Supplement: Supplementary file 1 — Supplementary Material 1: Supplementary Figure 1. Funnel plot for incidence of hypothyroidism following hemithyroidectomy. Supplementary Figure 2. Funnel plot for incidence of thyroxine supplementation following hemithyroidectomy. Supplementary Figure 3. Funnel plot for incidence of overt hypothyroidism following hemithyroidectomy. Supplementary Figure 4. Individual and pooled WMD for pre-operative TSH between hypothyroid and euthyroid groups following hemithyroidectomy. Supplementary Figure 5. Individual and pooled RR for pre-operative anti-TPO positivity between hypothyroid and euthyroid groups following hemithyroidectomy. Supplementary Figure 6. Individual and pooled RR for pre-operative anti-Tg positivity between hypothyroid and euthyroid groups following hemithyroidectomy. Supplementary Figure 7. Individual and pooled RR for right sided hemithyroidectomy between hypothyroid and euthyroid groups following hemithyroidectomy. Supplementary Figure 8. Individual and pooled RR for malignant pathology between hypothyroid and euthyroid groups following hemithyroidectomy. Supplementary Figure 9. Individual and pooled RR of postoperative hypothyroidism for patients with a family of thyroid dysfunction.Supplementary Figure 10. Individual and pooled WMD of BMI between hypothyroid and euthyroid groups. Supplementary Figure 11. Individual and pooled WMD of remnant thyroid volume between hypothyroid and euthyroid groups. Supplementary Figure 12. Funnel plot assessing asymmetry of WMD for age between hypothyroid and euthyroid groups. Supplementary Figure 13. Funnel plot assessing asymmetry of RR of female sex between hypothyroid and euthyroid groups. Supplementary Figure 14. Funnel plot assessing asymmetry of RR for Hashimoto’s thyroiditis between hypothyroid and euthyroid groups. Supplementary Figure 15. Funnel plot assessing asymmetry of WMD of pre-operative TSH between hypothyroid and euthyroid groups. Supplementary Figure 16. Funnel plot assessing asymmetry of RR for malig [file 13044_2024_200_MOESM1_ESM.zip › SF17SideFunnel.pdf]

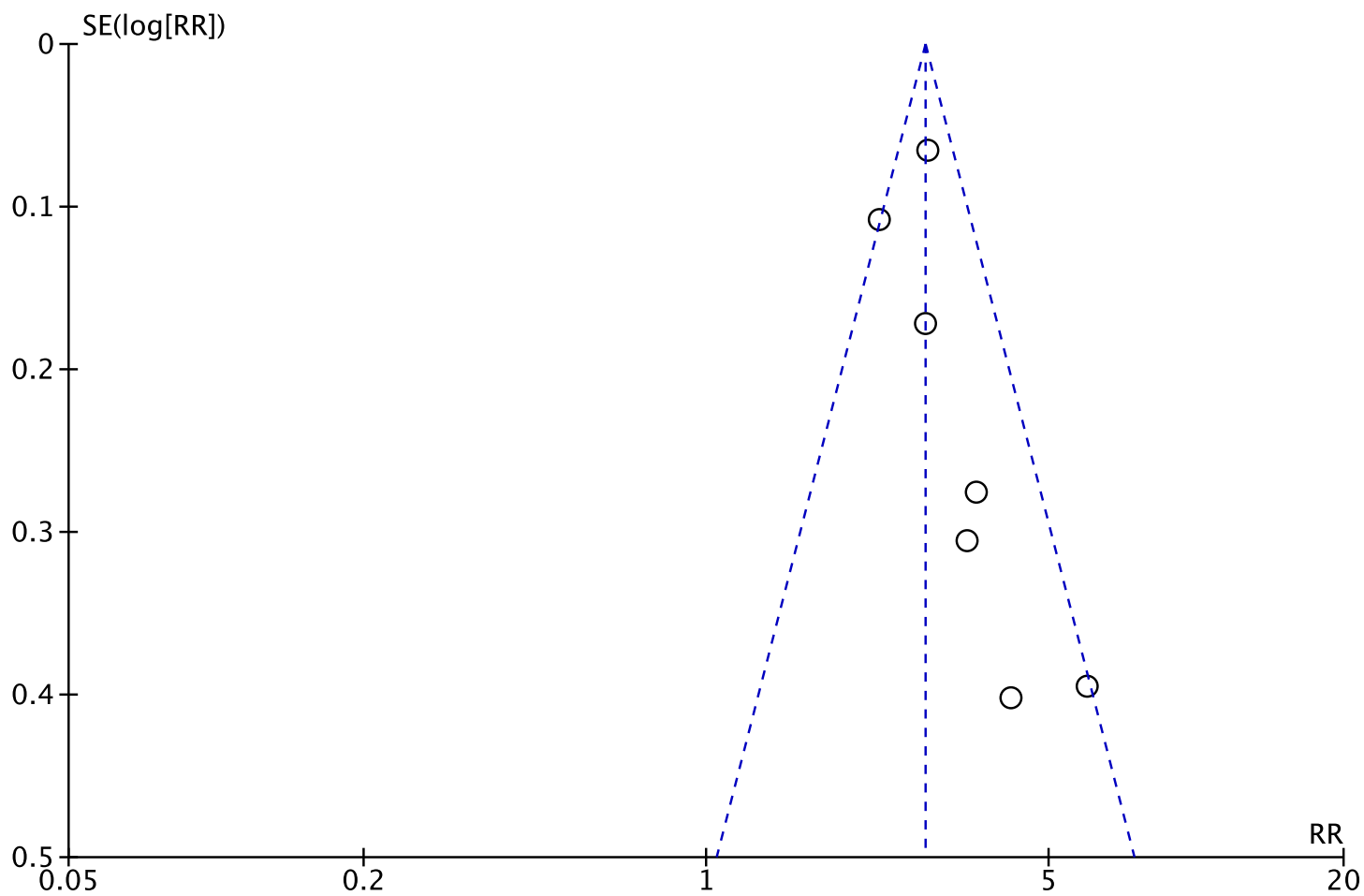

Supplement: Supplementary file 1 — Supplementary Material 1: Supplementary Figure 1. Funnel plot for incidence of hypothyroidism following hemithyroidectomy. Supplementary Figure 2. Funnel plot for incidence of thyroxine supplementation following hemithyroidectomy. Supplementary Figure 3. Funnel plot for incidence of overt hypothyroidism following hemithyroidectomy. Supplementary Figure 4. Individual and pooled WMD for pre-operative TSH between hypothyroid and euthyroid groups following hemithyroidectomy. Supplementary Figure 5. Individual and pooled RR for pre-operative anti-TPO positivity between hypothyroid and euthyroid groups following hemithyroidectomy. Supplementary Figure 6. Individual and pooled RR for pre-operative anti-Tg positivity between hypothyroid and euthyroid groups following hemithyroidectomy. Supplementary Figure 7. Individual and pooled RR for right sided hemithyroidectomy between hypothyroid and euthyroid groups following hemithyroidectomy. Supplementary Figure 8. Individual and pooled RR for malignant pathology between hypothyroid and euthyroid groups following hemithyroidectomy. Supplementary Figure 9. Individual and pooled RR of postoperative hypothyroidism for patients with a family of thyroid dysfunction.Supplementary Figure 10. Individual and pooled WMD of BMI between hypothyroid and euthyroid groups. Supplementary Figure 11. Individual and pooled WMD of remnant thyroid volume between hypothyroid and euthyroid groups. Supplementary Figure 12. Funnel plot assessing asymmetry of WMD for age between hypothyroid and euthyroid groups. Supplementary Figure 13. Funnel plot assessing asymmetry of RR of female sex between hypothyroid and euthyroid groups. Supplementary Figure 14. Funnel plot assessing asymmetry of RR for Hashimoto’s thyroiditis between hypothyroid and euthyroid groups. Supplementary Figure 15. Funnel plot assessing asymmetry of WMD of pre-operative TSH between hypothyroid and euthyroid groups. Supplementary Figure 16. Funnel plot assessing asymmetry of RR for malig [file 13044_2024_200_MOESM1_ESM.zip › SF18TSH2Funnel.pdf]

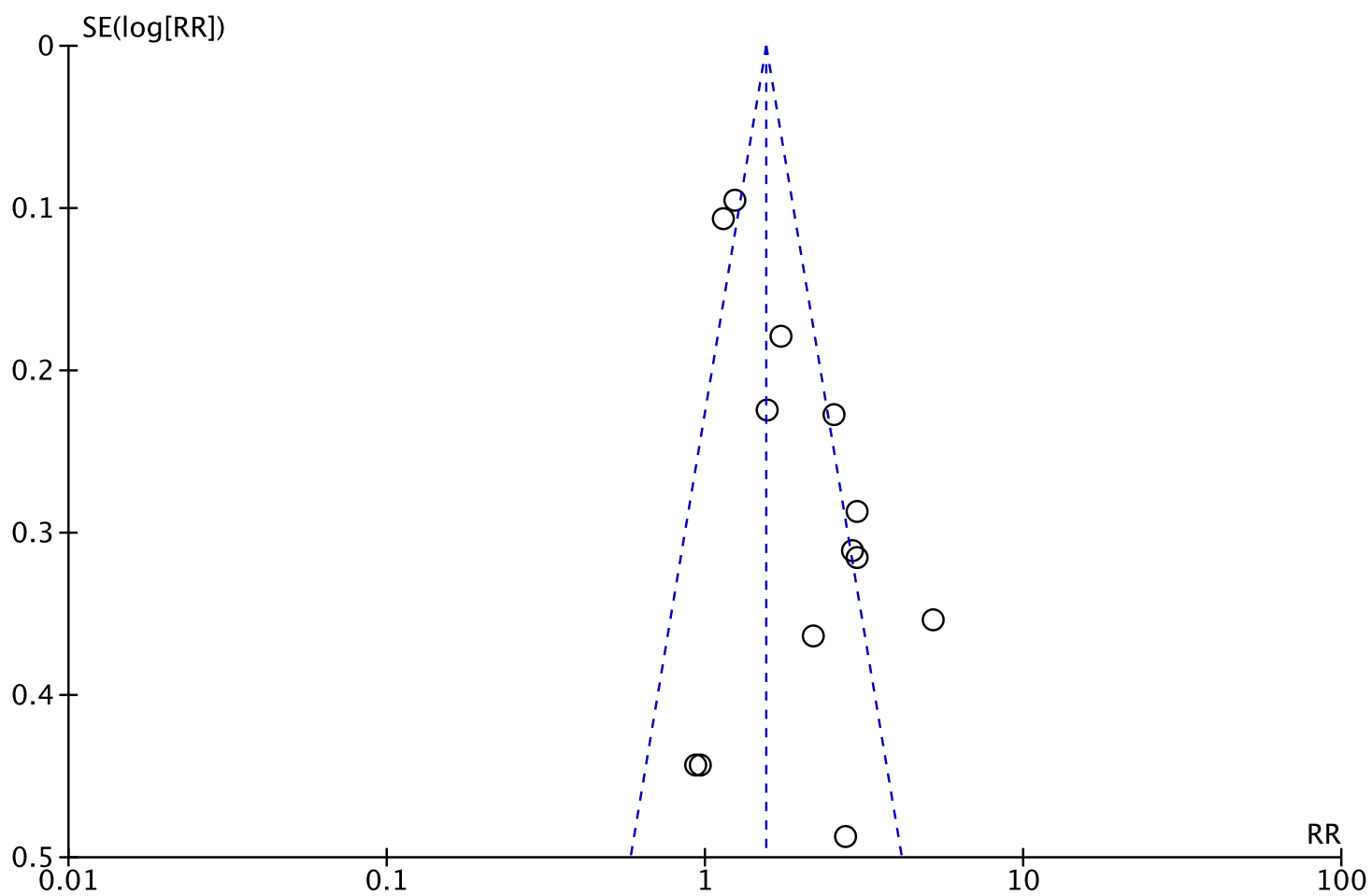

Supplement: Supplementary file 1 — Supplementary Material 1: Supplementary Figure 1. Funnel plot for incidence of hypothyroidism following hemithyroidectomy. Supplementary Figure 2. Funnel plot for incidence of thyroxine supplementation following hemithyroidectomy. Supplementary Figure 3. Funnel plot for incidence of overt hypothyroidism following hemithyroidectomy. Supplementary Figure 4. Individual and pooled WMD for pre-operative TSH between hypothyroid and euthyroid groups following hemithyroidectomy. Supplementary Figure 5. Individual and pooled RR for pre-operative anti-TPO positivity between hypothyroid and euthyroid groups following hemithyroidectomy. Supplementary Figure 6. Individual and pooled RR for pre-operative anti-Tg positivity between hypothyroid and euthyroid groups following hemithyroidectomy. Supplementary Figure 7. Individual and pooled RR for right sided hemithyroidectomy between hypothyroid and euthyroid groups following hemithyroidectomy. Supplementary Figure 8. Individual and pooled RR for malignant pathology between hypothyroid and euthyroid groups following hemithyroidectomy. Supplementary Figure 9. Individual and pooled RR of postoperative hypothyroidism for patients with a family of thyroid dysfunction.Supplementary Figure 10. Individual and pooled WMD of BMI between hypothyroid and euthyroid groups. Supplementary Figure 11. Individual and pooled WMD of remnant thyroid volume between hypothyroid and euthyroid groups. Supplementary Figure 12. Funnel plot assessing asymmetry of WMD for age between hypothyroid and euthyroid groups. Supplementary Figure 13. Funnel plot assessing asymmetry of RR of female sex between hypothyroid and euthyroid groups. Supplementary Figure 14. Funnel plot assessing asymmetry of RR for Hashimoto’s thyroiditis between hypothyroid and euthyroid groups. Supplementary Figure 15. Funnel plot assessing asymmetry of WMD of pre-operative TSH between hypothyroid and euthyroid groups. Supplementary Figure 16. Funnel plot assessing asymmetry of RR for malig [file 13044_2024_200_MOESM1_ESM.zip › SF19AntiTPOFunnel.pdf]

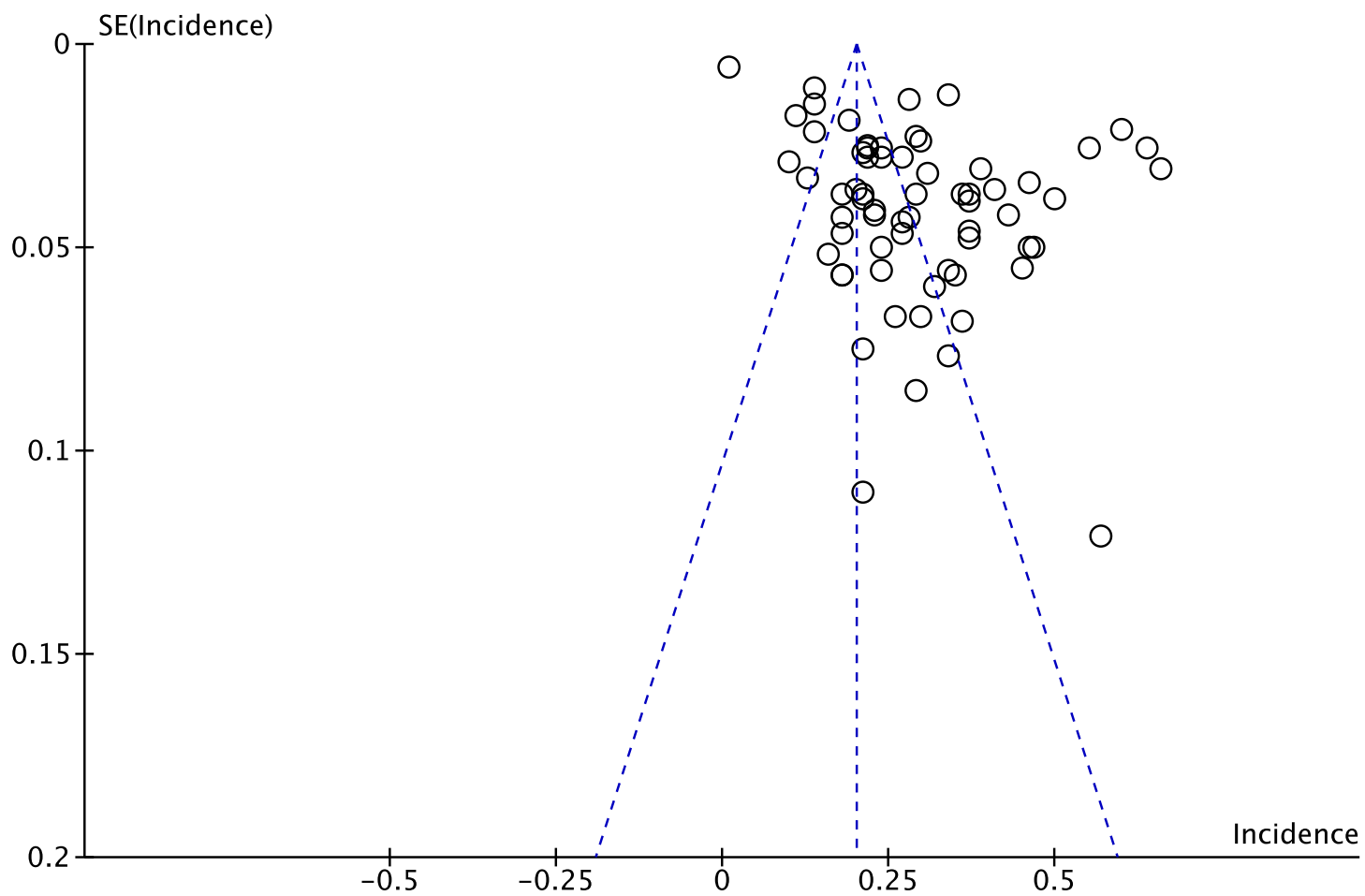

Supplement: Supplementary file 1 — Supplementary Material 1: Supplementary Figure 1. Funnel plot for incidence of hypothyroidism following hemithyroidectomy. Supplementary Figure 2. Funnel plot for incidence of thyroxine supplementation following hemithyroidectomy. Supplementary Figure 3. Funnel plot for incidence of overt hypothyroidism following hemithyroidectomy. Supplementary Figure 4. Individual and pooled WMD for pre-operative TSH between hypothyroid and euthyroid groups following hemithyroidectomy. Supplementary Figure 5. Individual and pooled RR for pre-operative anti-TPO positivity between hypothyroid and euthyroid groups following hemithyroidectomy. Supplementary Figure 6. Individual and pooled RR for pre-operative anti-Tg positivity between hypothyroid and euthyroid groups following hemithyroidectomy. Supplementary Figure 7. Individual and pooled RR for right sided hemithyroidectomy between hypothyroid and euthyroid groups following hemithyroidectomy. Supplementary Figure 8. Individual and pooled RR for malignant pathology between hypothyroid and euthyroid groups following hemithyroidectomy. Supplementary Figure 9. Individual and pooled RR of postoperative hypothyroidism for patients with a family of thyroid dysfunction.Supplementary Figure 10. Individual and pooled WMD of BMI between hypothyroid and euthyroid groups. Supplementary Figure 11. Individual and pooled WMD of remnant thyroid volume between hypothyroid and euthyroid groups. Supplementary Figure 12. Funnel plot assessing asymmetry of WMD for age between hypothyroid and euthyroid groups. Supplementary Figure 13. Funnel plot assessing asymmetry of RR of female sex between hypothyroid and euthyroid groups. Supplementary Figure 14. Funnel plot assessing asymmetry of RR for Hashimoto’s thyroiditis between hypothyroid and euthyroid groups. Supplementary Figure 15. Funnel plot assessing asymmetry of WMD of pre-operative TSH between hypothyroid and euthyroid groups. Supplementary Figure 16. Funnel plot assessing asymmetry of RR for malig [file 13044_2024_200_MOESM1_ESM.zip › SF1HypothyroidFunnel.pdf]

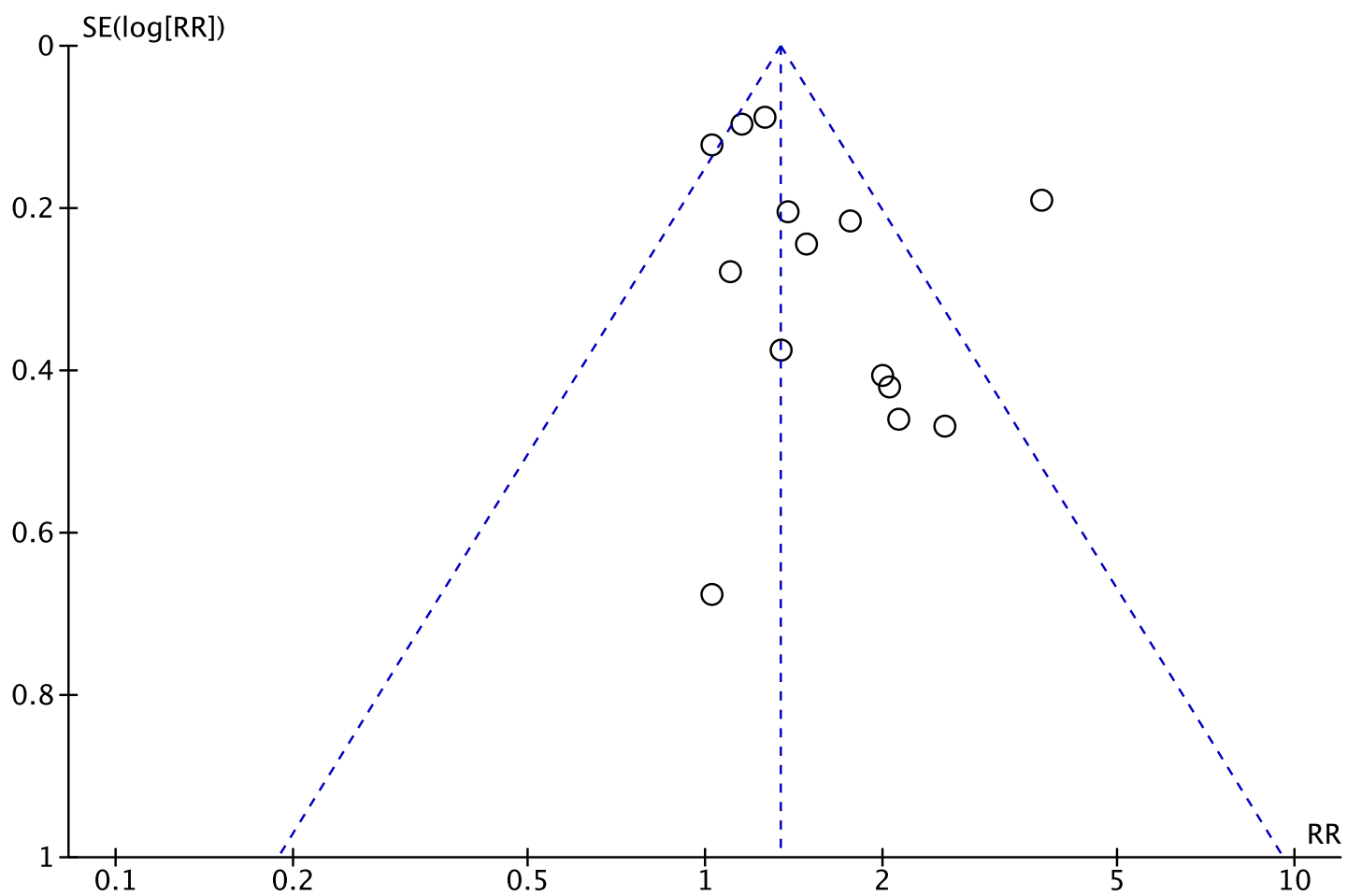

Supplement: Supplementary file 1 — Supplementary Material 1: Supplementary Figure 1. Funnel plot for incidence of hypothyroidism following hemithyroidectomy. Supplementary Figure 2. Funnel plot for incidence of thyroxine supplementation following hemithyroidectomy. Supplementary Figure 3. Funnel plot for incidence of overt hypothyroidism following hemithyroidectomy. Supplementary Figure 4. Individual and pooled WMD for pre-operative TSH between hypothyroid and euthyroid groups following hemithyroidectomy. Supplementary Figure 5. Individual and pooled RR for pre-operative anti-TPO positivity between hypothyroid and euthyroid groups following hemithyroidectomy. Supplementary Figure 6. Individual and pooled RR for pre-operative anti-Tg positivity between hypothyroid and euthyroid groups following hemithyroidectomy. Supplementary Figure 7. Individual and pooled RR for right sided hemithyroidectomy between hypothyroid and euthyroid groups following hemithyroidectomy. Supplementary Figure 8. Individual and pooled RR for malignant pathology between hypothyroid and euthyroid groups following hemithyroidectomy. Supplementary Figure 9. Individual and pooled RR of postoperative hypothyroidism for patients with a family of thyroid dysfunction.Supplementary Figure 10. Individual and pooled WMD of BMI between hypothyroid and euthyroid groups. Supplementary Figure 11. Individual and pooled WMD of remnant thyroid volume between hypothyroid and euthyroid groups. Supplementary Figure 12. Funnel plot assessing asymmetry of WMD for age between hypothyroid and euthyroid groups. Supplementary Figure 13. Funnel plot assessing asymmetry of RR of female sex between hypothyroid and euthyroid groups. Supplementary Figure 14. Funnel plot assessing asymmetry of RR for Hashimoto’s thyroiditis between hypothyroid and euthyroid groups. Supplementary Figure 15. Funnel plot assessing asymmetry of WMD of pre-operative TSH between hypothyroid and euthyroid groups. Supplementary Figure 16. Funnel plot assessing asymmetry of RR for malig [file 13044_2024_200_MOESM1_ESM.zip › SF20AntiTgFunnel.pdf]

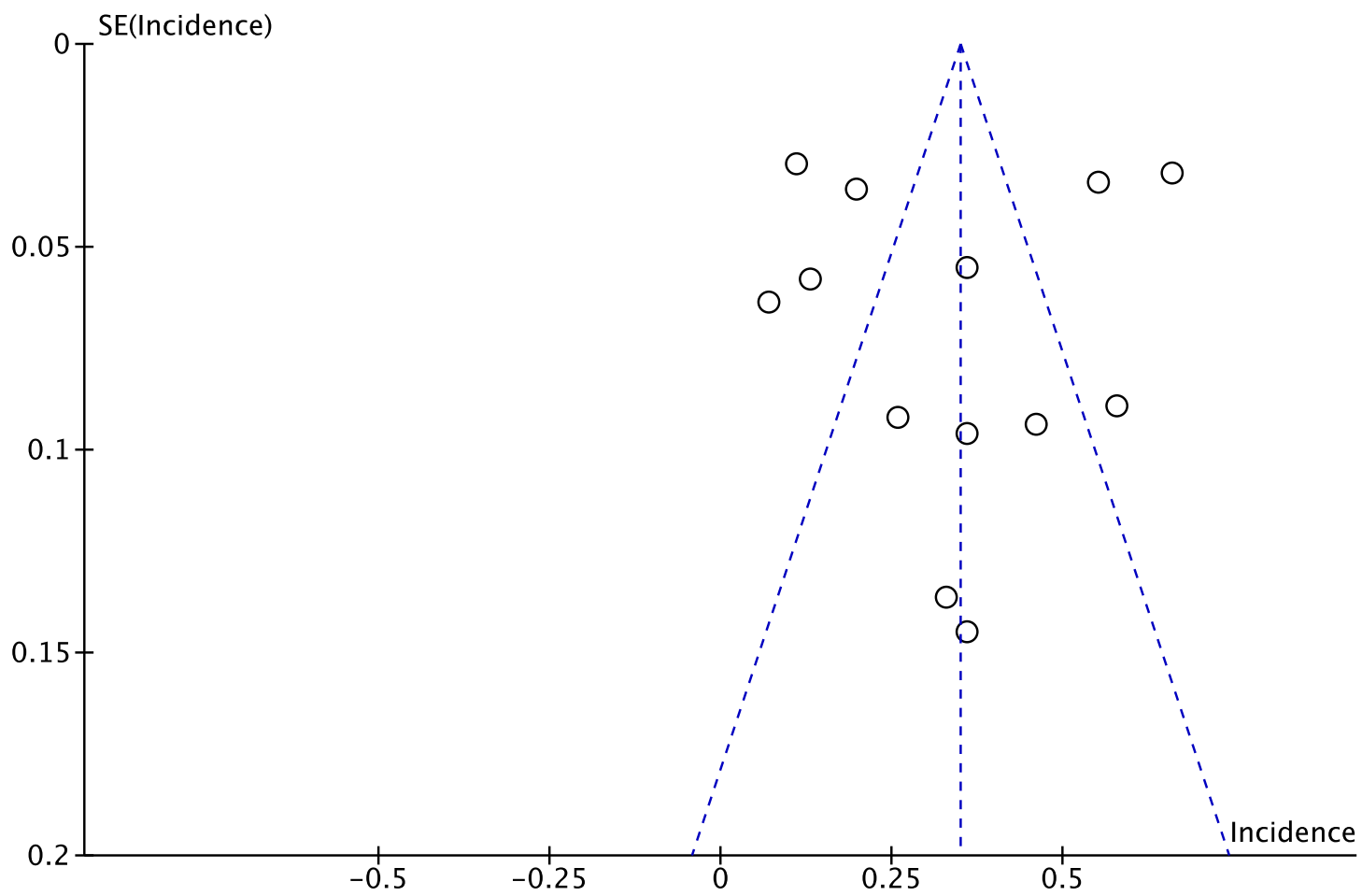

Supplement: Supplementary file 1 — Supplementary Material 1: Supplementary Figure 1. Funnel plot for incidence of hypothyroidism following hemithyroidectomy. Supplementary Figure 2. Funnel plot for incidence of thyroxine supplementation following hemithyroidectomy. Supplementary Figure 3. Funnel plot for incidence of overt hypothyroidism following hemithyroidectomy. Supplementary Figure 4. Individual and pooled WMD for pre-operative TSH between hypothyroid and euthyroid groups following hemithyroidectomy. Supplementary Figure 5. Individual and pooled RR for pre-operative anti-TPO positivity between hypothyroid and euthyroid groups following hemithyroidectomy. Supplementary Figure 6. Individual and pooled RR for pre-operative anti-Tg positivity between hypothyroid and euthyroid groups following hemithyroidectomy. Supplementary Figure 7. Individual and pooled RR for right sided hemithyroidectomy between hypothyroid and euthyroid groups following hemithyroidectomy. Supplementary Figure 8. Individual and pooled RR for malignant pathology between hypothyroid and euthyroid groups following hemithyroidectomy. Supplementary Figure 9. Individual and pooled RR of postoperative hypothyroidism for patients with a family of thyroid dysfunction.Supplementary Figure 10. Individual and pooled WMD of BMI between hypothyroid and euthyroid groups. Supplementary Figure 11. Individual and pooled WMD of remnant thyroid volume between hypothyroid and euthyroid groups. Supplementary Figure 12. Funnel plot assessing asymmetry of WMD for age between hypothyroid and euthyroid groups. Supplementary Figure 13. Funnel plot assessing asymmetry of RR of female sex between hypothyroid and euthyroid groups. Supplementary Figure 14. Funnel plot assessing asymmetry of RR for Hashimoto’s thyroiditis between hypothyroid and euthyroid groups. Supplementary Figure 15. Funnel plot assessing asymmetry of WMD of pre-operative TSH between hypothyroid and euthyroid groups. Supplementary Figure 16. Funnel plot assessing asymmetry of RR for malig [file 13044_2024_200_MOESM1_ESM.zip › SF21TransientFunnel.pdf]

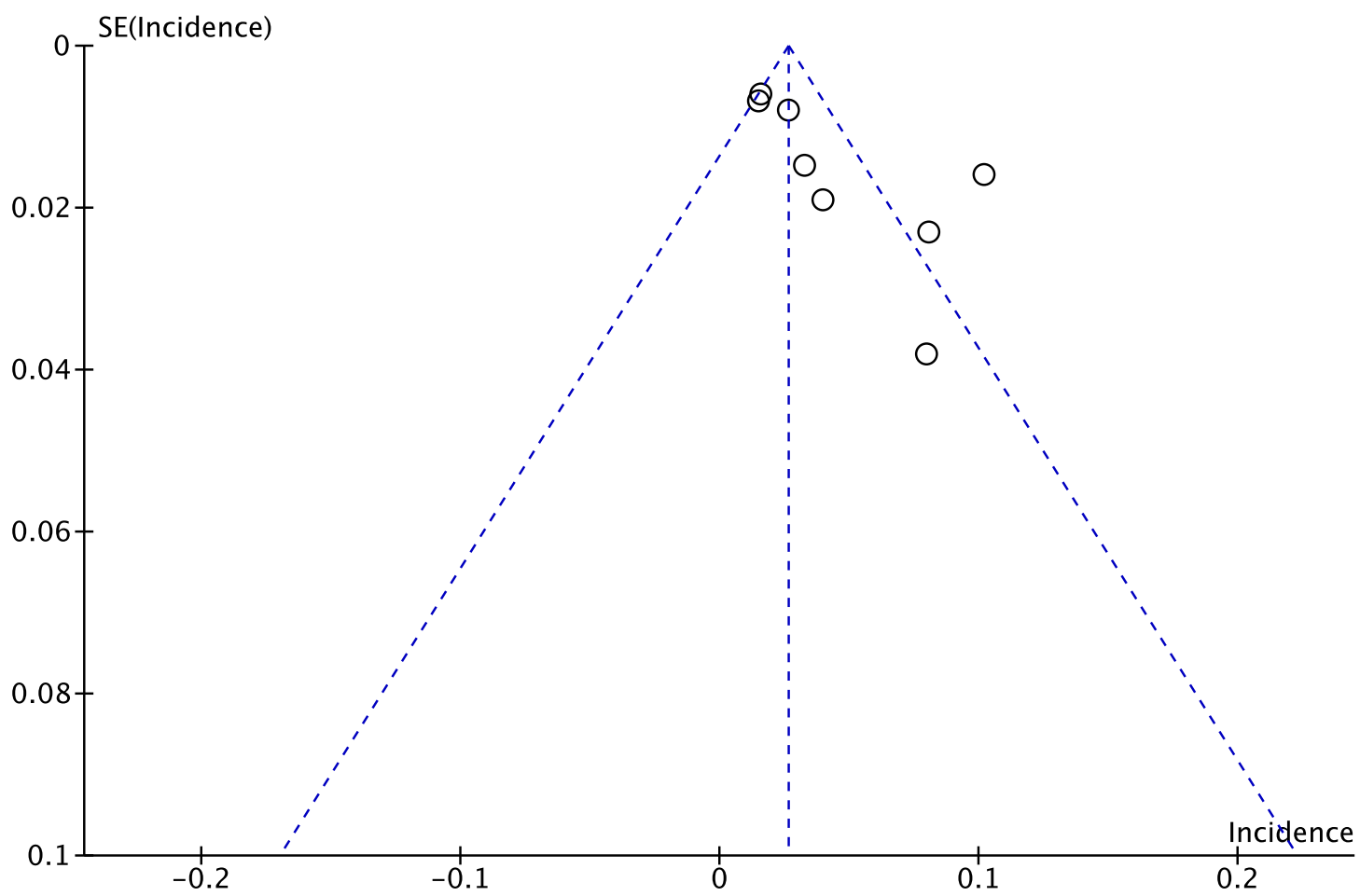

Supplement: Supplementary file 1 — Supplementary Material 1: Supplementary Figure 1. Funnel plot for incidence of hypothyroidism following hemithyroidectomy. Supplementary Figure 2. Funnel plot for incidence of thyroxine supplementation following hemithyroidectomy. Supplementary Figure 3. Funnel plot for incidence of overt hypothyroidism following hemithyroidectomy. Supplementary Figure 4. Individual and pooled WMD for pre-operative TSH between hypothyroid and euthyroid groups following hemithyroidectomy. Supplementary Figure 5. Individual and pooled RR for pre-operative anti-TPO positivity between hypothyroid and euthyroid groups following hemithyroidectomy. Supplementary Figure 6. Individual and pooled RR for pre-operative anti-Tg positivity between hypothyroid and euthyroid groups following hemithyroidectomy. Supplementary Figure 7. Individual and pooled RR for right sided hemithyroidectomy between hypothyroid and euthyroid groups following hemithyroidectomy. Supplementary Figure 8. Individual and pooled RR for malignant pathology between hypothyroid and euthyroid groups following hemithyroidectomy. Supplementary Figure 9. Individual and pooled RR of postoperative hypothyroidism for patients with a family of thyroid dysfunction.Supplementary Figure 10. Individual and pooled WMD of BMI between hypothyroid and euthyroid groups. Supplementary Figure 11. Individual and pooled WMD of remnant thyroid volume between hypothyroid and euthyroid groups. Supplementary Figure 12. Funnel plot assessing asymmetry of WMD for age between hypothyroid and euthyroid groups. Supplementary Figure 13. Funnel plot assessing asymmetry of RR of female sex between hypothyroid and euthyroid groups. Supplementary Figure 14. Funnel plot assessing asymmetry of RR for Hashimoto’s thyroiditis between hypothyroid and euthyroid groups. Supplementary Figure 15. Funnel plot assessing asymmetry of WMD of pre-operative TSH between hypothyroid and euthyroid groups. Supplementary Figure 16. Funnel plot assessing asymmetry of RR for malig [file 13044_2024_200_MOESM1_ESM.zip › SF2ThyroxineFunnel.pdf]

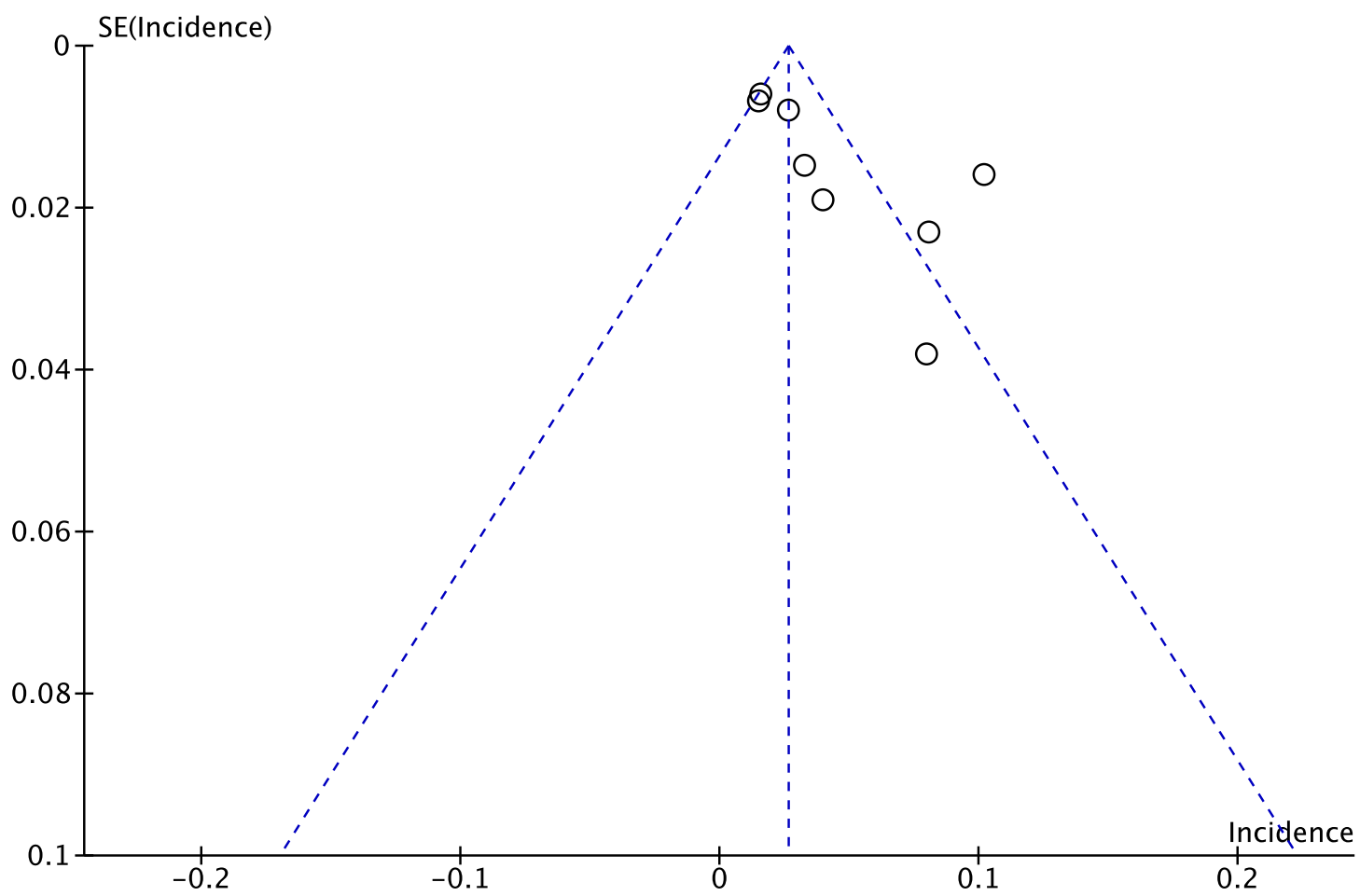

Supplement: Supplementary file 1 — Supplementary Material 1: Supplementary Figure 1. Funnel plot for incidence of hypothyroidism following hemithyroidectomy. Supplementary Figure 2. Funnel plot for incidence of thyroxine supplementation following hemithyroidectomy. Supplementary Figure 3. Funnel plot for incidence of overt hypothyroidism following hemithyroidectomy. Supplementary Figure 4. Individual and pooled WMD for pre-operative TSH between hypothyroid and euthyroid groups following hemithyroidectomy. Supplementary Figure 5. Individual and pooled RR for pre-operative anti-TPO positivity between hypothyroid and euthyroid groups following hemithyroidectomy. Supplementary Figure 6. Individual and pooled RR for pre-operative anti-Tg positivity between hypothyroid and euthyroid groups following hemithyroidectomy. Supplementary Figure 7. Individual and pooled RR for right sided hemithyroidectomy between hypothyroid and euthyroid groups following hemithyroidectomy. Supplementary Figure 8. Individual and pooled RR for malignant pathology between hypothyroid and euthyroid groups following hemithyroidectomy. Supplementary Figure 9. Individual and pooled RR of postoperative hypothyroidism for patients with a family of thyroid dysfunction.Supplementary Figure 10. Individual and pooled WMD of BMI between hypothyroid and euthyroid groups. Supplementary Figure 11. Individual and pooled WMD of remnant thyroid volume between hypothyroid and euthyroid groups. Supplementary Figure 12. Funnel plot assessing asymmetry of WMD for age between hypothyroid and euthyroid groups. Supplementary Figure 13. Funnel plot assessing asymmetry of RR of female sex between hypothyroid and euthyroid groups. Supplementary Figure 14. Funnel plot assessing asymmetry of RR for Hashimoto’s thyroiditis between hypothyroid and euthyroid groups. Supplementary Figure 15. Funnel plot assessing asymmetry of WMD of pre-operative TSH between hypothyroid and euthyroid groups. Supplementary Figure 16. Funnel plot assessing asymmetry of RR for malig [file 13044_2024_200_MOESM1_ESM.zip › SF3OvertFunnel.pdf]

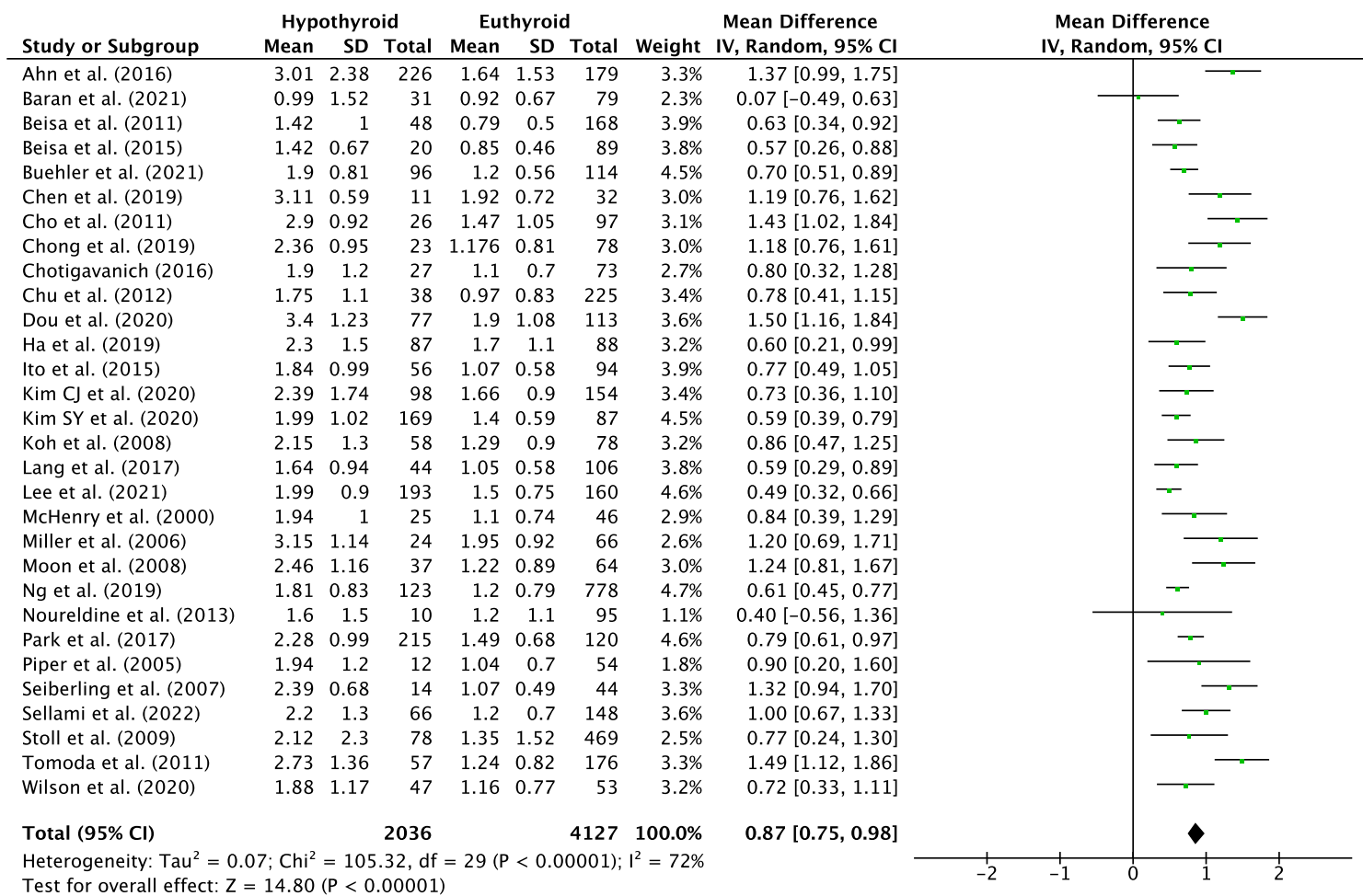

Supplement: Supplementary file 1 — Supplementary Material 1: Supplementary Figure 1. Funnel plot for incidence of hypothyroidism following hemithyroidectomy. Supplementary Figure 2. Funnel plot for incidence of thyroxine supplementation following hemithyroidectomy. Supplementary Figure 3. Funnel plot for incidence of overt hypothyroidism following hemithyroidectomy. Supplementary Figure 4. Individual and pooled WMD for pre-operative TSH between hypothyroid and euthyroid groups following hemithyroidectomy. Supplementary Figure 5. Individual and pooled RR for pre-operative anti-TPO positivity between hypothyroid and euthyroid groups following hemithyroidectomy. Supplementary Figure 6. Individual and pooled RR for pre-operative anti-Tg positivity between hypothyroid and euthyroid groups following hemithyroidectomy. Supplementary Figure 7. Individual and pooled RR for right sided hemithyroidectomy between hypothyroid and euthyroid groups following hemithyroidectomy. Supplementary Figure 8. Individual and pooled RR for malignant pathology between hypothyroid and euthyroid groups following hemithyroidectomy. Supplementary Figure 9. Individual and pooled RR of postoperative hypothyroidism for patients with a family of thyroid dysfunction.Supplementary Figure 10. Individual and pooled WMD of BMI between hypothyroid and euthyroid groups. Supplementary Figure 11. Individual and pooled WMD of remnant thyroid volume between hypothyroid and euthyroid groups. Supplementary Figure 12. Funnel plot assessing asymmetry of WMD for age between hypothyroid and euthyroid groups. Supplementary Figure 13. Funnel plot assessing asymmetry of RR of female sex between hypothyroid and euthyroid groups. Supplementary Figure 14. Funnel plot assessing asymmetry of RR for Hashimoto’s thyroiditis between hypothyroid and euthyroid groups. Supplementary Figure 15. Funnel plot assessing asymmetry of WMD of pre-operative TSH between hypothyroid and euthyroid groups. Supplementary Figure 16. Funnel plot assessing asymmetry of RR for malig [file 13044_2024_200_MOESM1_ESM.zip › SF4TSH.pdf]

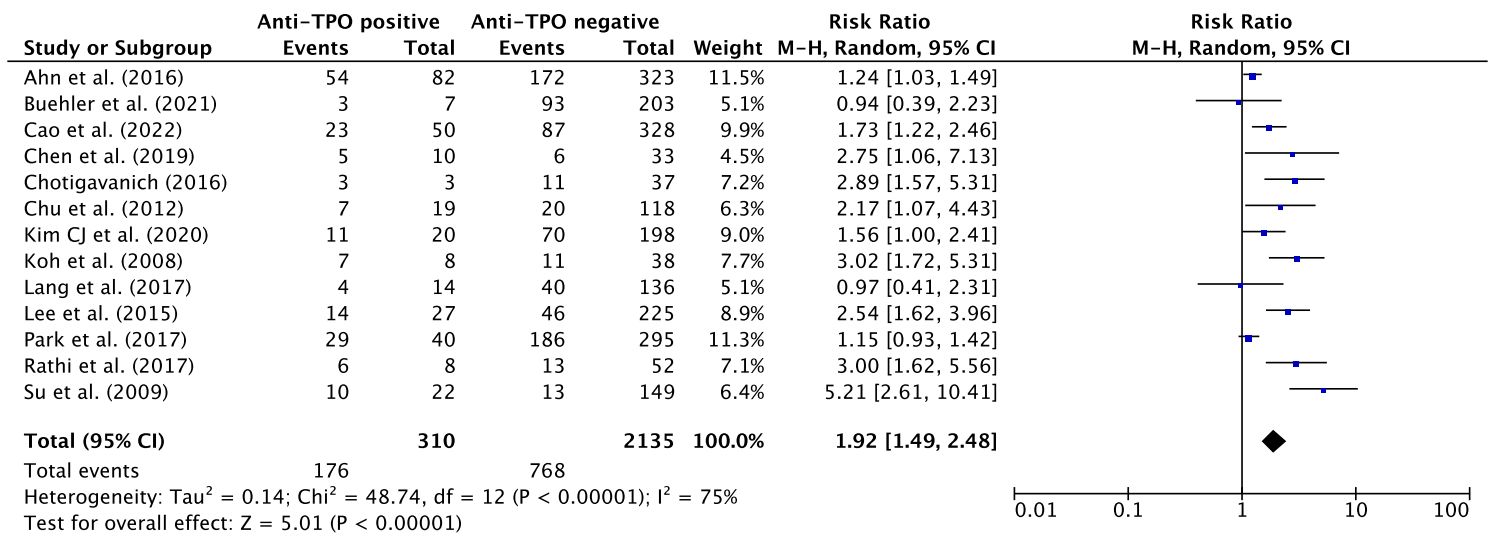

Supplement: Supplementary file 1 — Supplementary Material 1: Supplementary Figure 1. Funnel plot for incidence of hypothyroidism following hemithyroidectomy. Supplementary Figure 2. Funnel plot for incidence of thyroxine supplementation following hemithyroidectomy. Supplementary Figure 3. Funnel plot for incidence of overt hypothyroidism following hemithyroidectomy. Supplementary Figure 4. Individual and pooled WMD for pre-operative TSH between hypothyroid and euthyroid groups following hemithyroidectomy. Supplementary Figure 5. Individual and pooled RR for pre-operative anti-TPO positivity between hypothyroid and euthyroid groups following hemithyroidectomy. Supplementary Figure 6. Individual and pooled RR for pre-operative anti-Tg positivity between hypothyroid and euthyroid groups following hemithyroidectomy. Supplementary Figure 7. Individual and pooled RR for right sided hemithyroidectomy between hypothyroid and euthyroid groups following hemithyroidectomy. Supplementary Figure 8. Individual and pooled RR for malignant pathology between hypothyroid and euthyroid groups following hemithyroidectomy. Supplementary Figure 9. Individual and pooled RR of postoperative hypothyroidism for patients with a family of thyroid dysfunction.Supplementary Figure 10. Individual and pooled WMD of BMI between hypothyroid and euthyroid groups. Supplementary Figure 11. Individual and pooled WMD of remnant thyroid volume between hypothyroid and euthyroid groups. Supplementary Figure 12. Funnel plot assessing asymmetry of WMD for age between hypothyroid and euthyroid groups. Supplementary Figure 13. Funnel plot assessing asymmetry of RR of female sex between hypothyroid and euthyroid groups. Supplementary Figure 14. Funnel plot assessing asymmetry of RR for Hashimoto’s thyroiditis between hypothyroid and euthyroid groups. Supplementary Figure 15. Funnel plot assessing asymmetry of WMD of pre-operative TSH between hypothyroid and euthyroid groups. Supplementary Figure 16. Funnel plot assessing asymmetry of RR for malig [file 13044_2024_200_MOESM1_ESM.zip › SF5TPO.pdf]

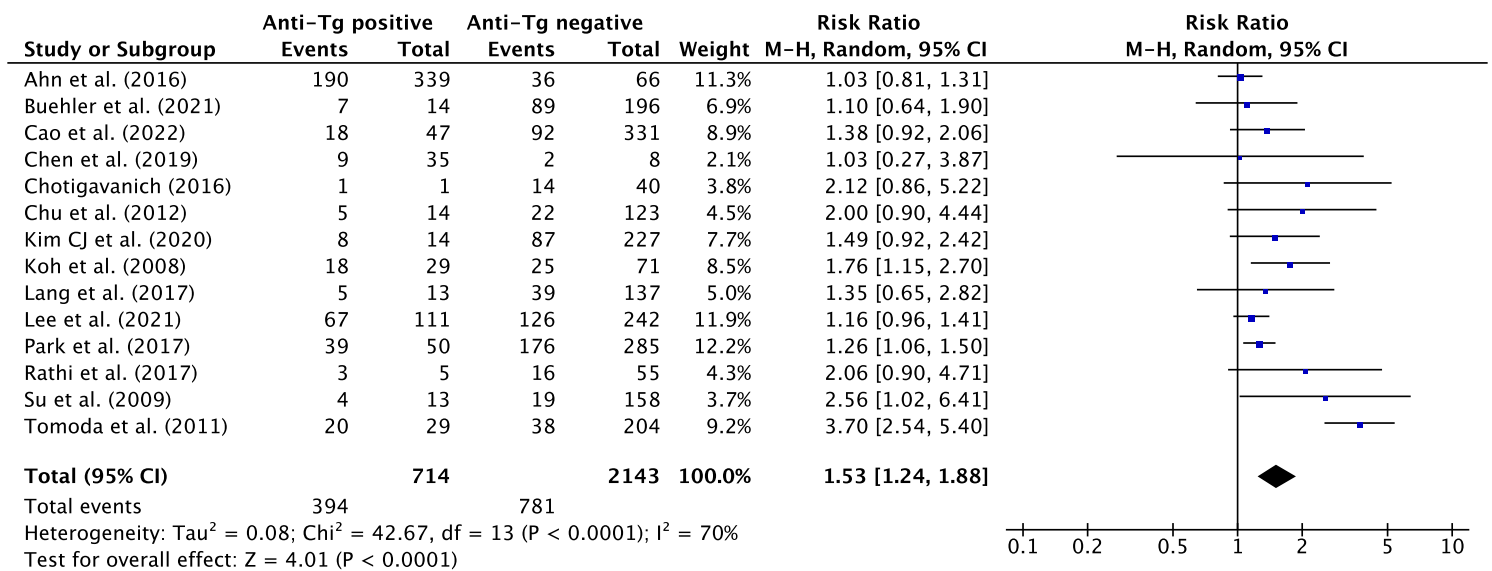

Supplement: Supplementary file 1 — Supplementary Material 1: Supplementary Figure 1. Funnel plot for incidence of hypothyroidism following hemithyroidectomy. Supplementary Figure 2. Funnel plot for incidence of thyroxine supplementation following hemithyroidectomy. Supplementary Figure 3. Funnel plot for incidence of overt hypothyroidism following hemithyroidectomy. Supplementary Figure 4. Individual and pooled WMD for pre-operative TSH between hypothyroid and euthyroid groups following hemithyroidectomy. Supplementary Figure 5. Individual and pooled RR for pre-operative anti-TPO positivity between hypothyroid and euthyroid groups following hemithyroidectomy. Supplementary Figure 6. Individual and pooled RR for pre-operative anti-Tg positivity between hypothyroid and euthyroid groups following hemithyroidectomy. Supplementary Figure 7. Individual and pooled RR for right sided hemithyroidectomy between hypothyroid and euthyroid groups following hemithyroidectomy. Supplementary Figure 8. Individual and pooled RR for malignant pathology between hypothyroid and euthyroid groups following hemithyroidectomy. Supplementary Figure 9. Individual and pooled RR of postoperative hypothyroidism for patients with a family of thyroid dysfunction.Supplementary Figure 10. Individual and pooled WMD of BMI between hypothyroid and euthyroid groups. Supplementary Figure 11. Individual and pooled WMD of remnant thyroid volume between hypothyroid and euthyroid groups. Supplementary Figure 12. Funnel plot assessing asymmetry of WMD for age between hypothyroid and euthyroid groups. Supplementary Figure 13. Funnel plot assessing asymmetry of RR of female sex between hypothyroid and euthyroid groups. Supplementary Figure 14. Funnel plot assessing asymmetry of RR for Hashimoto’s thyroiditis between hypothyroid and euthyroid groups. Supplementary Figure 15. Funnel plot assessing asymmetry of WMD of pre-operative TSH between hypothyroid and euthyroid groups. Supplementary Figure 16. Funnel plot assessing asymmetry of RR for malig [file 13044_2024_200_MOESM1_ESM.zip › SF6AntiTg.pdf]

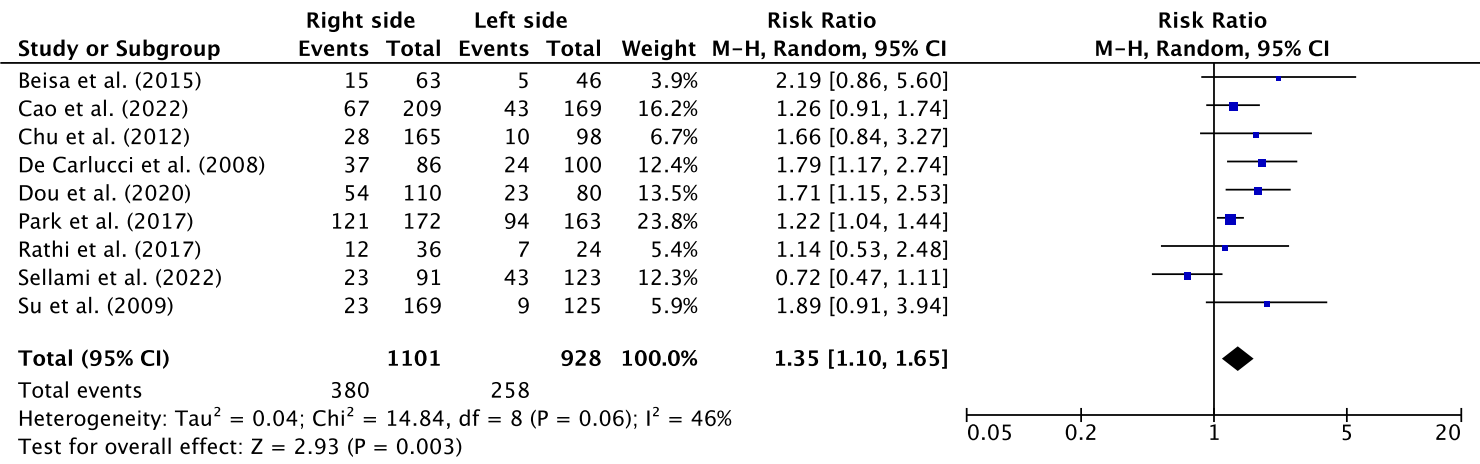

Supplement: Supplementary file 1 — Supplementary Material 1: Supplementary Figure 1. Funnel plot for incidence of hypothyroidism following hemithyroidectomy. Supplementary Figure 2. Funnel plot for incidence of thyroxine supplementation following hemithyroidectomy. Supplementary Figure 3. Funnel plot for incidence of overt hypothyroidism following hemithyroidectomy. Supplementary Figure 4. Individual and pooled WMD for pre-operative TSH between hypothyroid and euthyroid groups following hemithyroidectomy. Supplementary Figure 5. Individual and pooled RR for pre-operative anti-TPO positivity between hypothyroid and euthyroid groups following hemithyroidectomy. Supplementary Figure 6. Individual and pooled RR for pre-operative anti-Tg positivity between hypothyroid and euthyroid groups following hemithyroidectomy. Supplementary Figure 7. Individual and pooled RR for right sided hemithyroidectomy between hypothyroid and euthyroid groups following hemithyroidectomy. Supplementary Figure 8. Individual and pooled RR for malignant pathology between hypothyroid and euthyroid groups following hemithyroidectomy. Supplementary Figure 9. Individual and pooled RR of postoperative hypothyroidism for patients with a family of thyroid dysfunction.Supplementary Figure 10. Individual and pooled WMD of BMI between hypothyroid and euthyroid groups. Supplementary Figure 11. Individual and pooled WMD of remnant thyroid volume between hypothyroid and euthyroid groups. Supplementary Figure 12. Funnel plot assessing asymmetry of WMD for age between hypothyroid and euthyroid groups. Supplementary Figure 13. Funnel plot assessing asymmetry of RR of female sex between hypothyroid and euthyroid groups. Supplementary Figure 14. Funnel plot assessing asymmetry of RR for Hashimoto’s thyroiditis between hypothyroid and euthyroid groups. Supplementary Figure 15. Funnel plot assessing asymmetry of WMD of pre-operative TSH between hypothyroid and euthyroid groups. Supplementary Figure 16. Funnel plot assessing asymmetry of RR for malig [file 13044_2024_200_MOESM1_ESM.zip › SF7SideHemithyroidectomy.pdf]

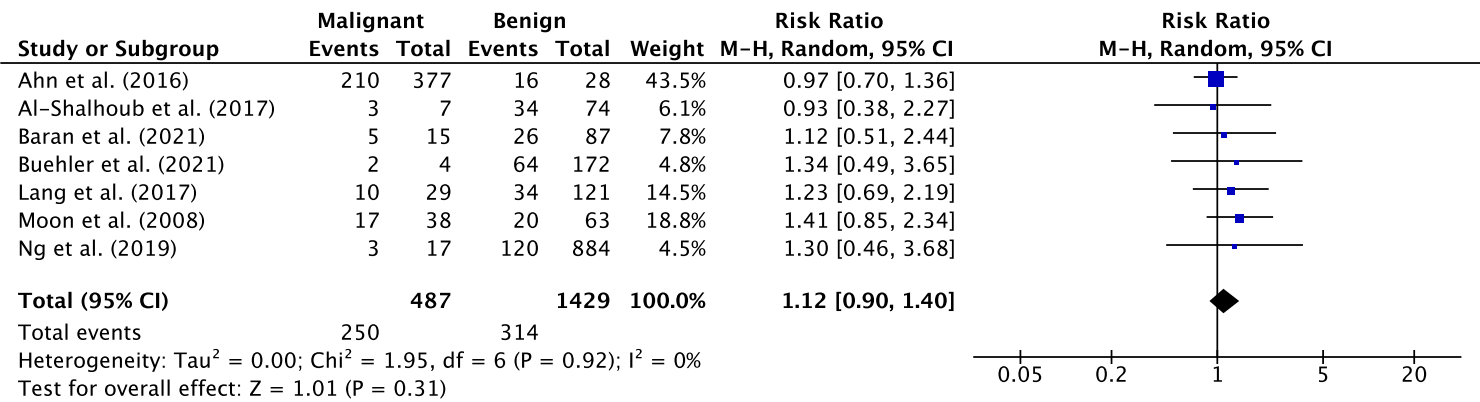

Supplement: Supplementary file 1 — Supplementary Material 1: Supplementary Figure 1. Funnel plot for incidence of hypothyroidism following hemithyroidectomy. Supplementary Figure 2. Funnel plot for incidence of thyroxine supplementation following hemithyroidectomy. Supplementary Figure 3. Funnel plot for incidence of overt hypothyroidism following hemithyroidectomy. Supplementary Figure 4. Individual and pooled WMD for pre-operative TSH between hypothyroid and euthyroid groups following hemithyroidectomy. Supplementary Figure 5. Individual and pooled RR for pre-operative anti-TPO positivity between hypothyroid and euthyroid groups following hemithyroidectomy. Supplementary Figure 6. Individual and pooled RR for pre-operative anti-Tg positivity between hypothyroid and euthyroid groups following hemithyroidectomy. Supplementary Figure 7. Individual and pooled RR for right sided hemithyroidectomy between hypothyroid and euthyroid groups following hemithyroidectomy. Supplementary Figure 8. Individual and pooled RR for malignant pathology between hypothyroid and euthyroid groups following hemithyroidectomy. Supplementary Figure 9. Individual and pooled RR of postoperative hypothyroidism for patients with a family of thyroid dysfunction.Supplementary Figure 10. Individual and pooled WMD of BMI between hypothyroid and euthyroid groups. Supplementary Figure 11. Individual and pooled WMD of remnant thyroid volume between hypothyroid and euthyroid groups. Supplementary Figure 12. Funnel plot assessing asymmetry of WMD for age between hypothyroid and euthyroid groups. Supplementary Figure 13. Funnel plot assessing asymmetry of RR of female sex between hypothyroid and euthyroid groups. Supplementary Figure 14. Funnel plot assessing asymmetry of RR for Hashimoto’s thyroiditis between hypothyroid and euthyroid groups. Supplementary Figure 15. Funnel plot assessing asymmetry of WMD of pre-operative TSH between hypothyroid and euthyroid groups. Supplementary Figure 16. Funnel plot assessing asymmetry of RR for malig [file 13044_2024_200_MOESM1_ESM.zip › SF8Malignant.pdf]

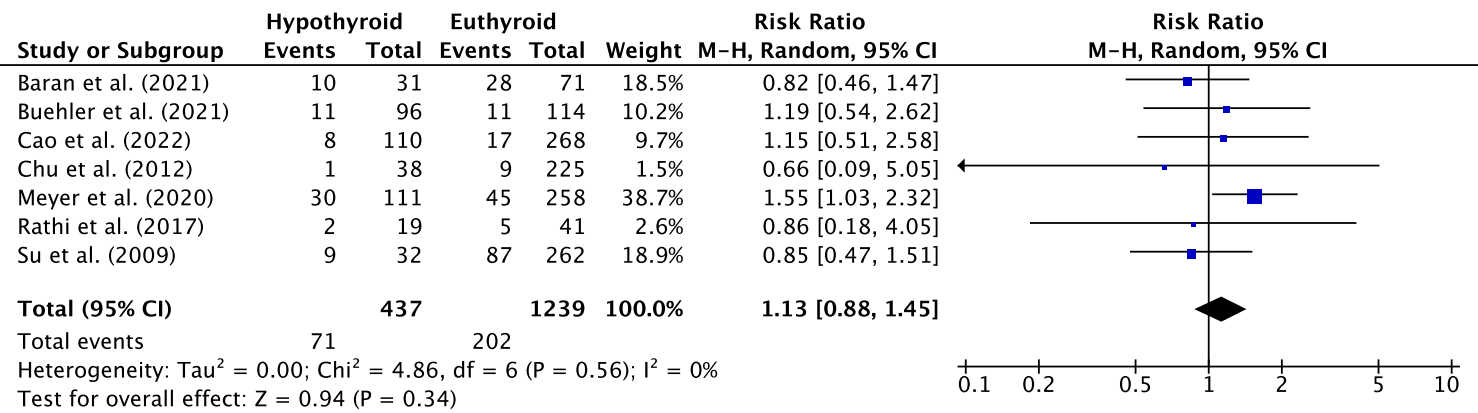

Supplement: Supplementary file 1 — Supplementary Material 1: Supplementary Figure 1. Funnel plot for incidence of hypothyroidism following hemithyroidectomy. Supplementary Figure 2. Funnel plot for incidence of thyroxine supplementation following hemithyroidectomy. Supplementary Figure 3. Funnel plot for incidence of overt hypothyroidism following hemithyroidectomy. Supplementary Figure 4. Individual and pooled WMD for pre-operative TSH between hypothyroid and euthyroid groups following hemithyroidectomy. Supplementary Figure 5. Individual and pooled RR for pre-operative anti-TPO positivity between hypothyroid and euthyroid groups following hemithyroidectomy. Supplementary Figure 6. Individual and pooled RR for pre-operative anti-Tg positivity between hypothyroid and euthyroid groups following hemithyroidectomy. Supplementary Figure 7. Individual and pooled RR for right sided hemithyroidectomy between hypothyroid and euthyroid groups following hemithyroidectomy. Supplementary Figure 8. Individual and pooled RR for malignant pathology between hypothyroid and euthyroid groups following hemithyroidectomy. Supplementary Figure 9. Individual and pooled RR of postoperative hypothyroidism for patients with a family of thyroid dysfunction.Supplementary Figure 10. Individual and pooled WMD of BMI between hypothyroid and euthyroid groups. Supplementary Figure 11. Individual and pooled WMD of remnant thyroid volume between hypothyroid and euthyroid groups. Supplementary Figure 12. Funnel plot assessing asymmetry of WMD for age between hypothyroid and euthyroid groups. Supplementary Figure 13. Funnel plot assessing asymmetry of RR of female sex between hypothyroid and euthyroid groups. Supplementary Figure 14. Funnel plot assessing asymmetry of RR for Hashimoto’s thyroiditis between hypothyroid and euthyroid groups. Supplementary Figure 15. Funnel plot assessing asymmetry of WMD of pre-operative TSH between hypothyroid and euthyroid groups. Supplementary Figure 16. Funnel plot assessing asymmetry of RR for malig [file 13044_2024_200_MOESM1_ESM.zip › SF9FamilyHx.pdf]
