# Supplementary material for: Hypothyroidism after hemithyroidectomy: a systematic review and meta-analysis
Source: Thyroid Res. 2024 Jul 8;17:18. doi: 10.1186/s13044-024-00200-z (PMC11229296; doi:10.1186/s13044-024-00200-z)
Supplement: Supplementary file 2 — Supplementary Material 2. [file 13044_2024_200_MOESM2_ESM.docx]

***Supplementary Table 1****: Modified Newcastle Ottawa Scale for risk of bias assessment.*

| **Study Name** | **Selection (/4)** | | | | **Comparability (/2)** | **Outcome (/3)** | | | **Total score (/6)** |
| --- | --- | --- | --- | --- | --- | --- | --- | --- | --- |
|  | **1) Representativeness of the exposed cohort** | **2) Selection of the non-exposed cohort*** | **3) Ascertainment of exposure** | **4) Demonstration that outcome of interest was not present at start of study** | **1) Comparability of cohorts on the basis of the design or analysis*** | **1) Assessment of outcome** | **2) Was follow-up long enough for outcomes to occur** | **3) Adequacy of follow up of cohorts** |  |
| **Abraham, C. R., et al. (2022).** | 1 | NA | 1 | 1 | NA | 1 | 1 | 1 | 6 |
| **Ahn, D., et al. (2016).** | 1 | NA | 1 | 1 | NA | 1 | 1 | 1 | 6 |
| **Akkari, M., et al. (2014).** | 0 | NA | 1 | 1 | NA | 1 | 1 | 0 | 4 |
| **Al-Shalhoub, A. K. and S. Al-Dhahri (2017).** | 1 | NA | 1 | 1 | NA | 1 | 1 | 0 | 5 |
| **Alsaleh, N., et al. (2021).** | 1 | NA | 1 | 0 | NA | 1 | 1 | 0 | 4 |
| **Antunes, C. M. and A. Taveira-Gomes (2013).** | 1 | NA | 1 | 0 | NA | 1 | 1 | 0 | 4 |
| **Attaallah, W., et al. (2015).** | 1 | NA | 1 | 0 | NA | 1 | 1 | 0 | 4 |
| **Balentine, C. J., et al. (2013).** | 1 | NA | 1 | 0 | NA | 1 | 0 | 0 | 3 |
| **Baran, J. A., et al. (2021).** | 0 | NA | 1 | 0 | NA | 1 | 1 | 0 | 3 |
| **Barczynski, M., et al. (2010).** | 1 | NA | 1 | 1 | NA | 1 | 1 | 1 | 6 |
| **Bauer, P. S., et al. (2013).** | 1 | NA | 1 | 1 | NA | 1 | 1 | 0 | 5 |
| **Beisa, V., et al. (2011).** | 1 | NA | 1 | 1 | NA | 1 | 1 | 0 | 5 |
| **Beisa, V., et al. (2015).** | 1 | NA | 1 | 1 | NA | 1 | 1 | 0 | 5 |
| **Berglund, J., et al. (1998).** | 1 | NA | 1 | 1 | NA | 1 | 1 | 1 | 6 |
| **Buehler, L. A., et al. (2021).** | 1 | NA | 1 | 1 | NA | 1 | 1 | 1 | 6 |
| **Chen, J., et al. (2019).** | 0 | NA | 1 | 1 | NA | 1 | 1 | 0 | 4 |
| **Cheung, P., et al. (1986).** | 1 | NA | 1 | 1 | NA | 1 | 1 | 0 | 5 |
| **Chidambaranathan, N., et al. (2021).** | 1 | NA | 1 | 0 | NA | 1 | 1 | 0 | 4 |
| **Cao, Z., et al. (2022).** | 1 | NA | 1 | 1 | NA | 1 | 0 | 1 | 5 |
| **Cho, M. J., et al. (2021).** | 1 | NA | 1 | 0 | NA | 1 | 1 | 1 | 5 |
| **Cho, J. S., et al. (2011).** | 1 | NA | 1 | 1 | NA | 1 | 1 | 0 | 5 |
| **Chong, S. S., et al. (2019).** | 1 | NA | 1 | 1 | NA | 1 | 1 | 0 | 5 |
| **Chotigavanich, C., et al. (2016).** | 1 | NA | 1 | 1 | NA | 1 | 0 | 0 | 4 |
| **Chu, K. K. W. and B. H. H. Lang (2012).** | 1 | NA | 1 | 1 | NA | 1 | 1 | 0 | 5 |
| **De Carlucci Jr, D., et al. (2008).** | 1 | NA | 1 | 1 | NA | 1 | 1 | 0 | 5 |
| **Dou, Y., et al. (2020).** | 1 | NA | 1 | 1 | NA | 1 | 1 | 1 | 6 |
| **Ergul, Z., et al. (2014).** | 1 | NA | 1 | 0 | NA | 1 | 1 | 0 | 4 |
| **Ha, T. K., et al. (2019).** | 1 | NA | 1 | 1 | NA | 1 | 0 | 0 | 4 |
| **Ito, M., et al. (2015).** | 1 | NA | 1 | 1 | NA | 1 | 1 | 0 | 5 |
| **Johner, A., et al. (2011).** | 1 | NA | 1 | 0 | NA | 1 | 0 | 0 | 3 |
| **Kim, C. J., et al. (2020).** | 1 | NA | 1 | 1 | NA | 1 | 1 | 0 | 5 |
| **Kim, S. Y., et al. (2020).** | 1 | NA | 1 | 1 | NA | 1 | 1 | 0 | 5 |
| **Koh, Y. W., et al. (2008).** | 1 | NA | 1 | 1 | NA | 1 | 1 | 0 | 5 |
| **Kristensen, T. T., et al. (2014).** | 1 | NA | 1 | 1 | NA | 1 | 1 | 0 | 5 |
| **Lang, B. H., et al. (2017).** | 1 | NA | 1 | 1 | NA | 1 | 1 | 0 | 5 |
| **Lankarani, M., et al. (2008).** | 1 | NA | 1 | 0 | NA | 1 | 1 | 1 | 5 |
| **Latoo, M. A., et al. (2020).** | 1 | NA | 1 | 1 | NA | 1 | 1 | 0 | 5 |
| **Lee, D. Y., et al. (2015).** | 1 | NA | 1 | 1 | NA | 1 | 1 | 0 | 5 |
| **Lee, S. J., et al. (2021).** | 1 | NA | 1 | 1 | NA | 1 | 1 | 0 | 5 |
| **Lindblom, P., et al. (2001).** | 1 | NA | 1 | 1 | NA | 1 | 0 | 0 | 4 |
| **McHenry, C. R. and S. J. Slusarczyk (2000).** | 1 | NA | 1 | 1 | NA | 1 | 1 | 0 | 5 |
| **Meyer, C. D., et al. (2020).** | 1 | NA | 1 | 1 | NA | 1 | 1 | 0 | 5 |
| **Miller, F. R., et al. (2006).** | 1 | NA | 1 | 1 | NA | 1 | 1 | 0 | 5 |
| **Moon, H. G., et al. (2008).** | 1 | NA | 1 | 0 | NA | 1 | 1 | 0 | 4 |
| **Morris, L., et al. (2013).** | 1 | NA | 1 | 0 | NA | 1 | 0 | 0 | 3 |
| **Ng, P., et al. (2019).** | 1 | NA | 1 | 1 | NA | 1 | 1 | 0 | 5 |
| **Noureldine, S. I., et al. (2013).** | 1 | NA | 1 | 0 | NA | 1 | 0 | 0 | 3 |
| **Park, S., et al. (2017).** | 1 | NA | 1 | 1 | NA | 1 | 1 | 0 | 5 |
| **Phitayakorn, R., et al. (2009).** | 1 | NA | 1 | 0 | NA | 1 | 1 | 0 | 4 |
| **Piper, H. G., et al. (2005).** | 1 | NA | 1 | 1 | NA | 1 | 0 | 1 | 5 |
| **Rathi A., S. D., Prasad B. (2017).** | 1 | NA | 1 | 1 | NA | 1 | 0 | 0 | 4 |
| **Said, M., et al. (2013).** | 1 | NA | 1 | 1 | NA | 1 | 1 | 1 | 6 |
| **Salih, A. M. (2018).** | 1 | NA | 1 | 1 | NA | 1 | 1 | 1 | 6 |
| **Sancho, J., et al. (2012).** | 1 | NA | 1 | 1 | NA | 1 | 0 | 0 | 4 |
| **Sarkis, L. M., et al. (2017).** | 1 | NA | 1 | 0 | NA | 1 | 0 | 0 | 3 |
| **Seiberling, K. A., et al. (2007).** | 1 | NA | 1 | 1 | NA | 1 | 0 | 0 | 4 |
| **Sellami, M., et al. (2022).** | 1 | NA | 1 | 1 | NA | 1 | 0 | 0 | 4 |
| **Spanheimer, P. M., et al. (2011).** | 1 | NA | 1 | 0 | NA | 1 | 0 | 0 | 3 |
| **Stoll, S. J., et al. (2009).** | 1 | NA | 1 | 1 | NA | 1 | 1 | 1 | 6 |
| **Su, S. Y., et al. (2009).** | 1 | NA | 1 | 1 | NA | 1 | 1 | 0 | 5 |
| **Tomoda, C., et al. (2011).** | 1 | NA | 1 | 1 | NA | 1 | 1 | 0 | 5 |
| **Vaiman, M., et al. (2008).** | 1 | NA | 1 | 0 | NA | 1 | 1 | 1 | 5 |
| **Wadström, C., et al. (1999).** | 1 | NA | 1 | 0 | NA | 0 | 1 | 1 | 4 |
| **Wilson, M., et al. (2020).** | 1 | NA | 1 | 1 | NA | 1 | 1 | 0 | 5 |
| **Wormald, R., et al. (2008).** | 1 | NA | 1 | 1 | NA | 1 | 1 | 1 | 6 |
| **Yetkin, G., et al. (2010).** | 1 | NA | 1 | 0 | NA | 1 | 1 | 1 | 5 |

*‘1’ denotes criteria met, ‘0’ criteria not met, ‘*’ fields that were not applicable due to no comparator arm with the study.*

***Supplementary Table 2****: Patient factors associated with transient hypothyroidism.*

| **Studies** | **Significant factors** | **Insignificant factors** |
| --- | --- | --- |
| **Ahn 2016 (**[**6**](#_ENREF_6)**)** | Age <46 (P=0.017), male (P=0.007), pre-operative TSH <2.6 (P=0.002) | Anti-TPO, Hashimoto thyroiditis |
| **Park 2017 (**[**5**](#_ENREF_5)**)** | Pre-operative TSH <= 1.7 (P=0.01), postoperative maximal TSH <= 7.1 (P<0.001) | Sex, age |
| **Buehler 2021 (**[**12**](#_ENREF_12)**)** | Lobectomy + isthmusectomy v lobectomy (P=0.049) | Age, sex, pre-operative TSH |
| **Dou 2021 (**[**7**](#_ENREF_7)**)** | Pre-operative TSH (P=0.014) | Age, sex, HT, others |
